# Supplementary material for: E2F1 acetylation directs p300/CBP-mediated histone acetylation at DNA double-strand breaks to facilitate repair
Source: Nat Commun. 2019 Oct 30;10:4951. doi: 10.1038/s41467-019-12861-8 (PMC6821830; doi:10.1038/s41467-019-12861-8)
Supplement: Supplementary file 1 — Supplementary Information [file 41467_2019_12861_MOESM1_ESM.pdf]

Supplementary Information

E2F1 Acetylation Directs p300/CBP-mediated Histone Acetylation at DNA Double-Strand Breaks to Facilitate Repair.

Manickavinayaham et al.

Supplementary Information contains

Supplementary Figures 1 – 7

Supplementary Tables 1 – 3

Supplementary Figure 1

a

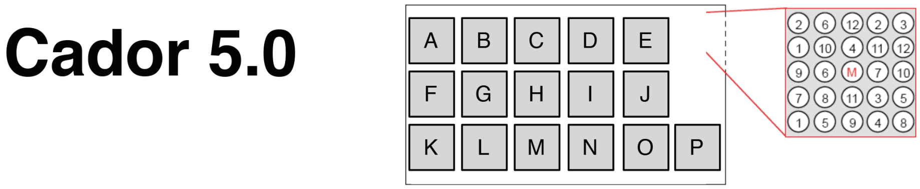

TUDOR<br>A1 TDRD2(NP_006853)<br>A2 TDRD3(Q9H7E2)<br>A3 TDRD4-1(Q9NUY9)<br>A4 TDRD4-2(Q9NUY9)<br>A5 TDRD4-3(Q9NUY9)<br>A6 TDRD7-1(NP_055105)<br>A7 TDRD7-3(NP_055105)<br>A8 EBNA-2Co-A(NP_055205)<br>A9 Ret-bp1(AAB28543)<br>A10 M96(AAH10013)<br>A11 53BP1(1-2)(NP_005648)<br>A12 53BP1(1)(NP_005648)	TUDOR +<br>B1 2C(NP_055876)<br>B2 RBP1 like-2 (NP_112739)<br>B3 SMN (NP_075012)<br>B4 CG1-72(NP_057102)<br>B5 PHF20(NP_057520)<br>B6 PHF20 MBT+TDR(NP_057520)<br>B7 Pombe 1(CAA22823)<br>B8 JMJD2A-2(NP_055478)<br>B9 JMJD2A1-2(NP_055478)<br>B10 LBR TDR(NP_919424)<br>B11 LBR211(NP_919424)<br>B12 SPF30(c)(O75940)	TUDOR / Tudor-Like<br>C1 Lin9 TDR(b)(AAH65302)<br>C2 JMJN 2B WT(f)(NP_055830)<br>C3 ARI4A (e)(NM_002892)<br>C4 PHF19(NP_001009936)<br>C5 SND1 (e)(NM_014390)<br>C6 UHRF1 Tudor-like(a)<br>C7 C420 Tudor-Like(p)<br>C8 C460 Tudor-Like(p)	MBT<br>D1 SFMBT F.L(j)<br>D2 SFMBt 4xMBT(j)<br>D3 L(3)MBT(NP_056293)<br>D4 L(3)MBT1-3(NP_056293)<br>D5 SCMH1(AAH21252)<br>D6 SCML1(NP_057413)<br>D7 SCML2(AAH64617)<br>D8 LML2(Q969R5)<br>D9 PHF20 MBT(NP_057520)<br>D10 CG1-72-MBT(NP_057102)	His-MBTs / WD40<br>E1 SFMBT1(a)<br>E2 SFMBT2(a)<br>E3 L3MBTL1(a)<br>E4 L3MBTL2(a)<br>E5 L3MBTL3(a)<br>E6 SCMH1(a)<br>E7 WD40 -WDR5(d)(NP_543124)<br>E8 WD40 -WDR9(NM_018963)<br>E9 WD40 -RbAb46(d)(BT007309)<br>E10 WD40 -RbAb48(d)(X74262)<br>E11 WD40 -HIRA(CR456503)<br>E12 WD40 -Mep50(d)(AF478464)	
PhD +<br>F1 BPTF(P+B)(BAA89208)<br>F2 ING2(e)(AAH50003)<br>F3 PHF2(NP_005383)<br>F4 PHF8(CAI42860)<br>F5 DATF1(CAI95708)<br>F6 Rag2(NM_000536)<br>F7 PCCX1(NP_055408)<br>F8 P300(P+B)(NM_001429)<br>F9 PHF20 PHD(NP_057520)<br>F10 PHD PHF3<br>F11 PHD PHF5<br>F12 PHD CHD5 (1-2)	PhD<br>G1 Dnmt3a-His/GST(g)<br>G2 Dnmt3b-His/GST(g)<br>G3 DnMT3L N-term-His/GST(g)<br>G4 Trim24 Brd-PhD(l)<br>G5 ING3(e) (NP_061944)<br>G6 ING4(e) (NP_001121054)<br>G7 ING5(e) (NP_115705)<br>G8 PHD _TIF1A(e) (O15164)<br>G9 TRI66(e) (O15016)<br>G10 BRPF1(e) (P55201)<br>G11 MLL4(e) (Q9UMN6)<br>G12 MTF2(e) (Q9Y483)	PhD +<br>H1 JMJD2APhD+2Tudor(NP_055478)<br>H2 JMJCPhD(NP_055478)<br>H3 M96Tudor+PhD(AAH10013)<br>H4 MYST4PhD+PhD(AAH48199)<br>H5 NSD1PhD+PWWP(Q96L73)<br>H6 WHSC1PhD+PWWP(NP_579877)<br>H7 BS69PhD+ BRD(AAH12586)<br>H8 ATRX<br>H9 RAL1<br>H10 BAZ1b/WSTF<br>H11 CBP<br>H12 TAF3 (k)	BROMO<br>I1 GCN5(Q92830)<br>I2 TAF1- D1(NP_620278)<br>I3 TAF1- D2(NP_620278)<br>I4 P/CAF (S71788)<br>I5 SNF2 beta(S45252)<br>I6 BAF180 1-2(NP_060635)<br>I7 BAF180-3(NP_060635)<br>I8 BAF180 3-4(NP_060635)<br>I9 BAF180 5-6(NP_060635)<br>I10 KAP-1(AAB37341)<br>I11 P300(NP_004371)<br>I12 WDR9 2(Q9NSI6)	BROMO /SANT / TSN<br>J1 Bromo-BAZ(NP_075381)<br>J2 Bromo-BRDT 1(AAH62700)<br>J3 SANT-MPP11-like(XP_379909)<br>J4 SANT-N-CoR2-2(Q9Y618)<br>J5 SANT-RERE(AAH62342)<br>J6 SANT-ADA2(NP_001479)<br>J7 SANT-Zuotin Rel.(XP_168590)<br>J8 TSN-p100(o) (NP_055205)<br>J9 TSN-p100 m5(o) (NP_055205)<br>J10 TSN-p100 m6(o) (NP_055205)	
CHROMO<br>K1 TIP60(h)(AAB18236)<br>K2 CHD2(h)(AAB87382)<br>K3 CHD4(h)(AAH38596)<br>K4 MPP8(h) (NP_059990)<br>K5 SMARCC2(h)(AAH26222)<br>K6 MRG15(h)(AAD29872)<br>K7 RBBP1(h)(AAD41239)<br>K8 PC2(h)(AAB80718)<br>K9 PC3(h)(AAG09180)<br>K10 CHD5(h)(AAK56405)<br>K11 CHD7 (1-2)(AB037837)<br>K12 CBX6/NPCD(BC012111)	CHROMO<br>L1 MI-2(h)(CAA60384)<br>L2 HP1alpha(h)(P45973)<br>L3 HP1gamma(h)(NP_057671)<br>L4 Msl3-like(h)(AAD38499)<br>L5 SUV39H1(h)(AAB92224)<br>L6 CBX1/HP1beta(h)(AAD21972)<br>L7 HP1 beta(h)(P23197)<br>L8 CDY1(h)(AAD22735)<br>L9 CHD1(e) (NP_001261)<br>L10 CBX4/PC2(e) (NM_003655)<br>L11 CBX7/RP4(e) (NM_175709)<br>L12 CBX5/HP1alpha(e)NM_012117)	CHROMO / BRK /MRG<br>M1 CBX3(e) (NM_016587)<br>M2 CBX2(e) (NM_005189)<br>M3 CDYL2(e) (NM_152342)<br>M4 CBX8(e) (NM_020649)<br>M5 BRK_SMCA2 (e)(NM_003070)<br>M6 BRK_SMCA4(e) (NM_003072)<br>M7 BRK_CHD6(e) (NM_032221)<br>M8 BRK_CHD7 (e)(NM_017780)<br>M9 BRK_Q6DTK9 (e)(NM_025134)<br>M10 MRG_MS3L1(e) (NM_078629)<br>M11 MRG_MO4L1(e) (NM_206839)	PWWP<br>N1 BRPF1(AAH53851)<br>N3 DNMT3B(Q9UBC3)<br>N4 HDGF (P51858)<br>N5 HRP-3(BAA90477)<br>N6 MSH6(P52701)<br>N7 NSD1(Q96L73)<br>N8 WHSC1-1(NP_579877)<br>N9 PSIP1(e) (NM_033222<br>N10 BRD1(e) (NM_014577<br>N11 ZCPW1 (e)(AL136735<br>N12 MBD5 (e)(NM_018328	PWWP / CW / SWIRM<br>O1 PWWP_PKCB1(e) (NM_183047<br>O2 PWWP_HDGR3(e) (NM_016073)<br>O3 PWWP_DNM3A (e) (NM_175629)<br>O4 CW3(AAH02725)<br>O5 CW5(BAA09485)<br>O6 CW6(XP_087384)<br>O7 SWIRM_ KIAA1915(BAB67808)<br>O8 SWIRM_KIAA0601(CAB72299)<br>O9 SWIRM_ADA2(NP_001479)	ANK<br>Q1 ANK-BARD1 (NP_000456)<br>Q2 ANK-GLP(m) (AAM09024.1)<br>Q3 ANK-Notch (NP_060087)<br>Q4 ANK-IKB alpha F.L. (e)<br>Other<br>Q5 TULP1<br>Q6 MeCP2(i)<br>Q7 PHD_ ZFP-1 His/GST(n)

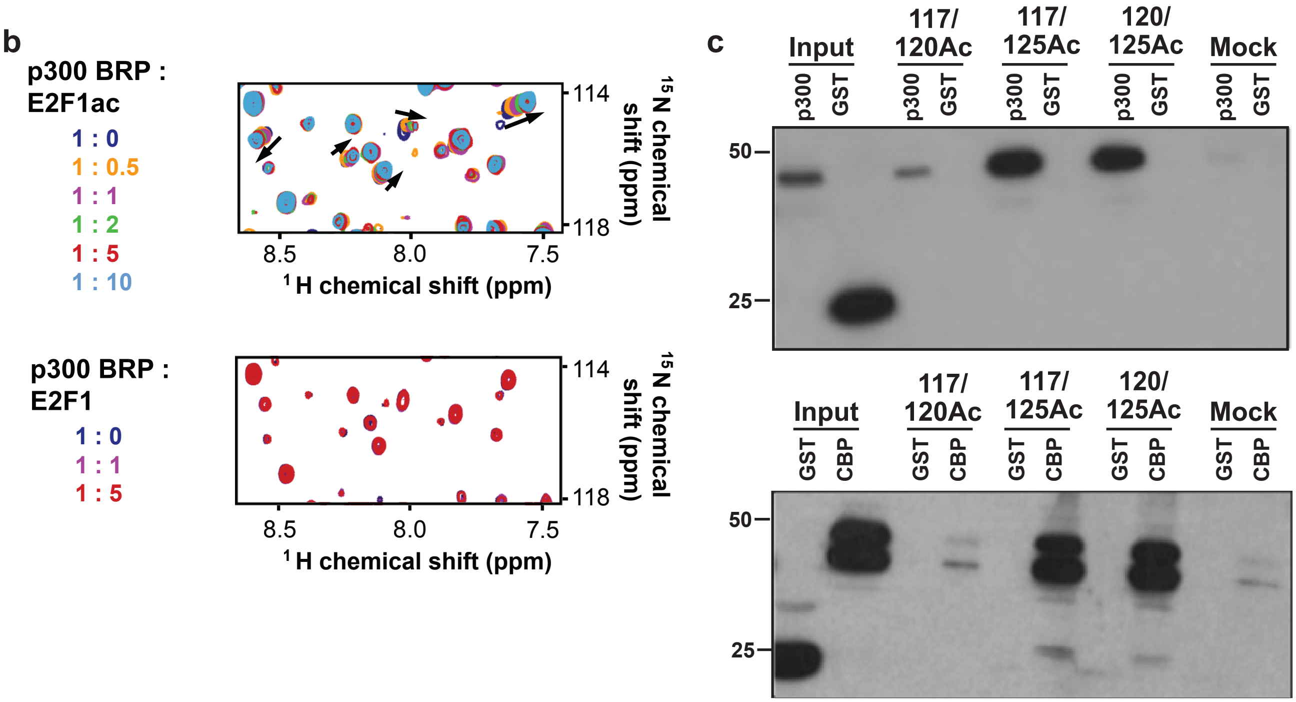

Supplementary Figure 1: Binding of acetylated E2F1 with p300 and CBP. (a) Layout of Cador 5.0 protein domain microarray, which contains 174 GST recombinant domain proteins. These proteins have been separated into blocks with each block consisting of up to 12 domain proteins printed in duplicate. Each block also contains a GST control located in the middle (M). **(b)** ^{1}H, ^{15}N heteronuclear single quantum coherence (HSQC) NMR titration experiments were performed using a recombinant GST-p300 fusion protein containing the bromo, the ring finger and PHD domains (BRP) and E2F1 peptides that were either acetylated (top) or unacetylated (bottom). **(c)** Biotinylated E2F1 peptides di-acetylated at K117/120, K117/125, or K120/125 were used as bait to pull down purified GST-fusion proteins containing the bromodomain domain of p300 (top) or CBP (bottom). Following pull down, western blot for GST was performed to examine binding between p300 or CBP and the E2F1 peptides. Source data of **c** is provided as Supplementary Data 5.

Supplementary Figure 2

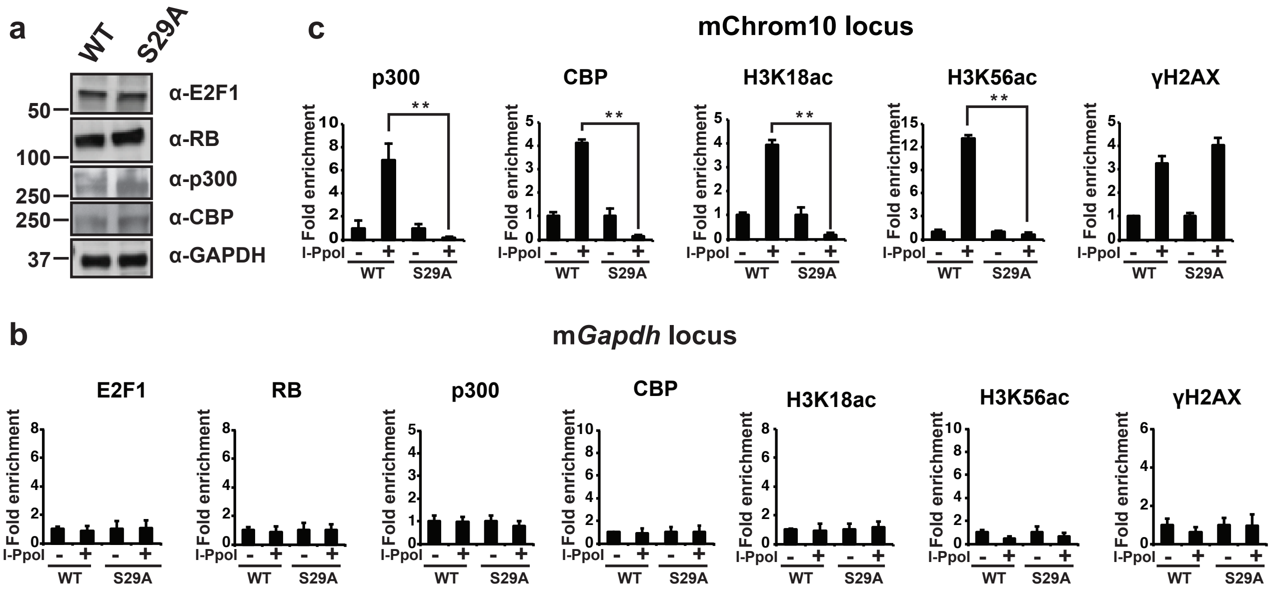

Supplementary Figure 2: Recruitment status of p300 and CBP to control and break sites. (a) Western blot analysis for E2F1, RB, p300, CBP and GAPDH was performed using whole cell extracts from primary wild type (WT) and *E2f1*$^{S29A/S29A}$ (S29A) MEFs. **(b)** The I-PpoI ChIP assay was performed using primary wild type (WT) and *E2f1*$^{S29A/S29A}$ (S29A) MEFs, uninfected (-) or infected (+) with a retrovirus expressing HA-ER*-I-PpoI as indicated, and induced with 4-OHT for 12h. The occupancy of the indicated factors was determined at the *Gapdh* locus. **(c)** The same I-PpoI ChIP assay was performed and analyzed as above and occupancy for the indicated proteins was determined for a region on mouse chromosome 10 (mChrom10), 269 bp 5' to an I-PpoI cut site. All graphs represent average ± SD of three independent experiments (n=3). P values were calculated by unpaired Student's *t*-test. (**) P ≤ 0.01 is highly significant. Source data of **a** is provided as Supplementary Data 5. Raw data of **b** and **c** are in Source Data File.

Supplementary Figure 3

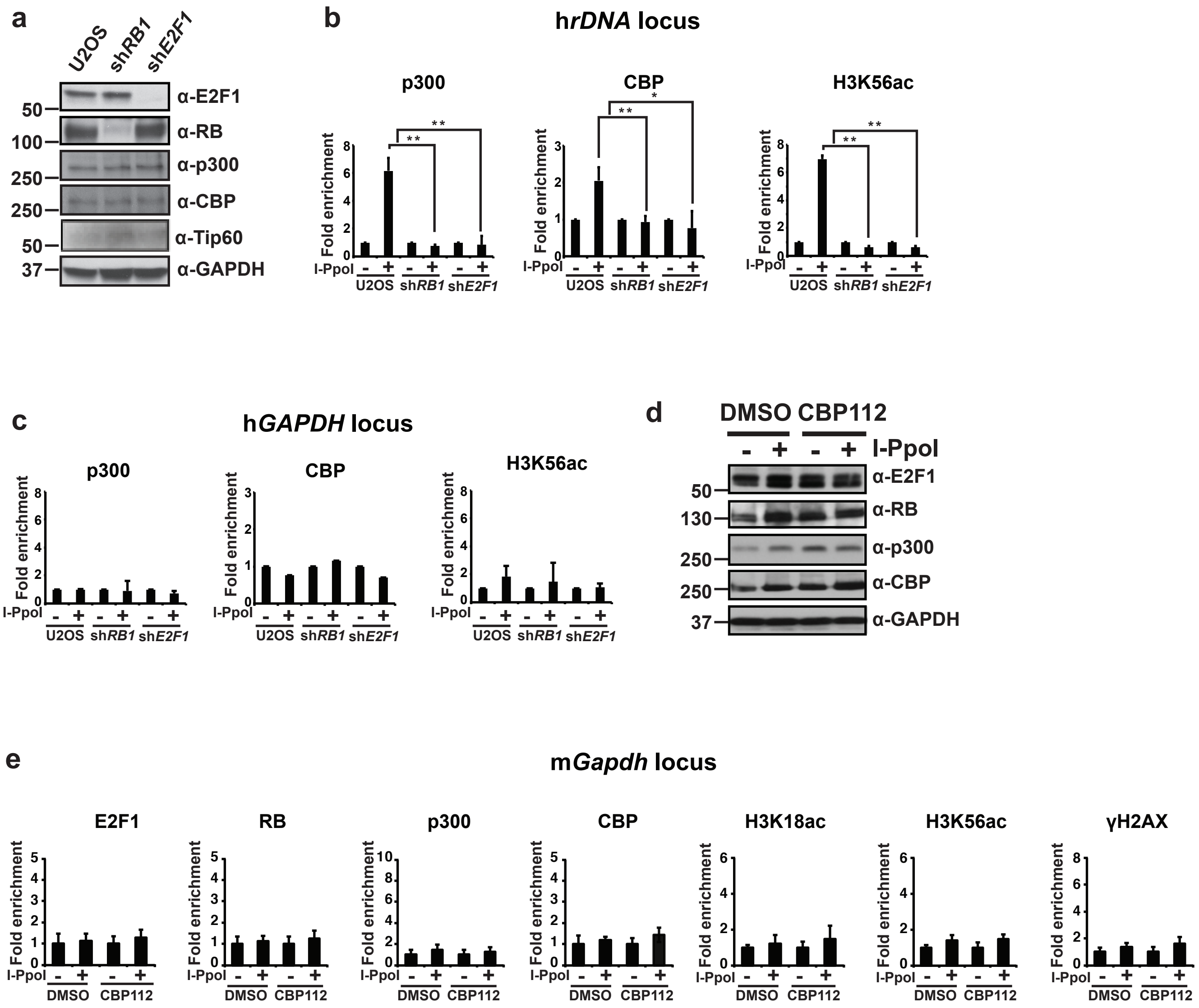

Supplementary Figure 3: Enrichment of proteins to control and DSB sites. (a) Western blot analysis for E2F1, RB, p300, CBP, Tip60 and GAPDH was performed using whole cell extracts from parental U2OS cells or U2OS cells depleted for RB (*shRB1*) or E2F1 (*shE2F1*). **(b and c)** The I-PpoI ChIP assay was performed using parental U2OS cells or U2OS cells depleted for RB (*shRB1*) or E2F1 (*shE2F1*), uninfected (-) or infected (+) with a retrovirus expressing HA-ER*-I-PpoI as indicated, and induced with 4-OHT for 12h. The occupancy of the indicated factors was determined at the *rDNA* locus **(b)** and *GAPDH* locus **(c)**. **(d)** Western blot analysis for E2F1, RB, p300, CBP and GAPDH was performed using whole cell extracts from primary wild type MEFs, untreated (DMSO vehicle) or treated with the CBP112 bromodomain inhibitor compound (1μM for 24h), and either minus (-) or plus (+) I-PpoI induction as indicated. **(e)** The I-PpoI ChIP assay was performed in primary wild type (WT) MEFs as indicated above, either with DMSO or CBP112 treatment. The occupancy of the indicated factors was determined at the *Gapdh* locus. All graphs represent average ± SD of three independent experiments (n=3). P values were calculated by unpaired Student's *t*-test. (**) P ≤ 0.01 is highly significant. (*) P ≤ 0.05 is significant. Source data of **d** is provided as Supplementary Data 5. Raw data of **b**, **c** and **e** are in Source Data File.

Supplementary Figure 4

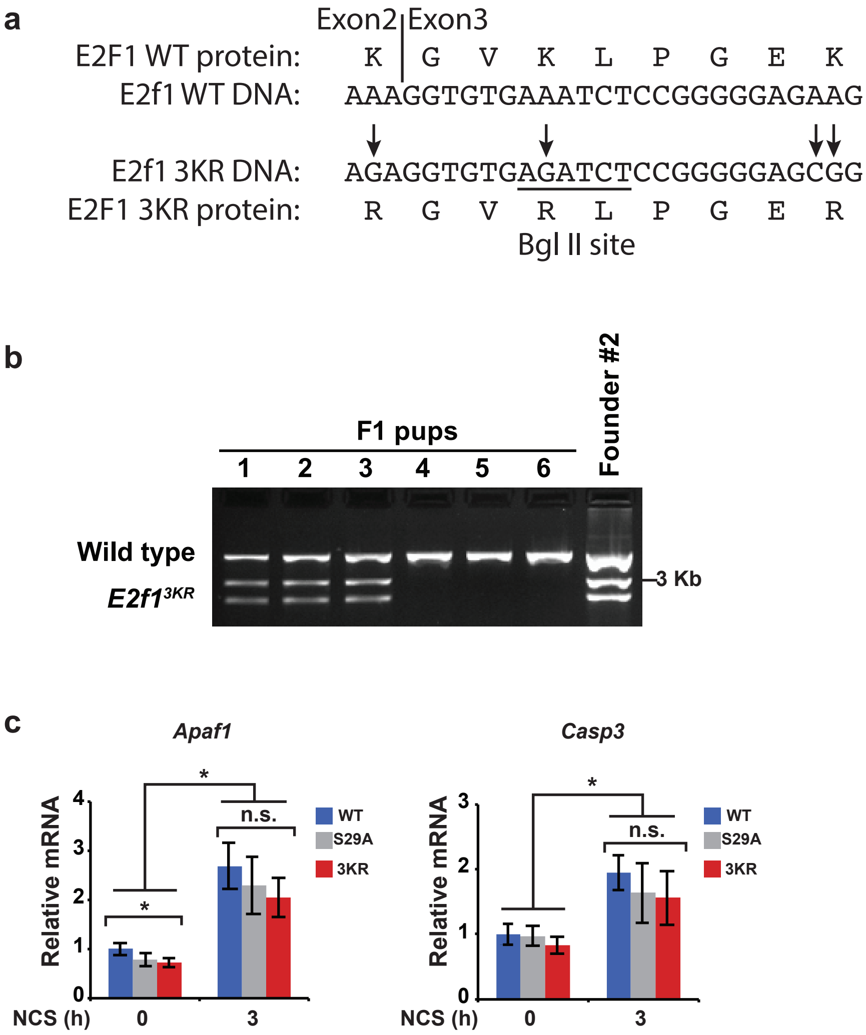

Supplementary Figure 4: Generation of an E2F1 3KR knock-in mouse model. (a) DNA and protein sequences of wild type and 3KR knock-in *E2f1* alleles with targeted mutations indicated. **(b)** Genotyping of founder *E2f1*^{3KR} mouse (#2) and F1 generation offspring by PCR amplification of *E2f1* genomic region spanning exons 2 and 3 followed by digestion with Bgl II. Heterozygous founder and F1 pups having both wild type and 3KR knock-in alleles and homozygous F1 pups having only wild type allele are shown by agarose gel electrophoresis. **(c)** Primary wild type (WT), *E2f1*$^{S29A/S29A}$ and *E2f1*$^{3KR/3KR}$ (3KR) MEFs were untreated or treated with NCS (250 ng per ml) for 3h. Total RNA was isolated and cDNA was prepared by RT-PCR. Expression of *Apaf1* and *Caspase3* (*Casp3*) was determined by qPCR of three independent experiments (n=3) and represented as average ± SD. P values were calculated by unpaired Student's *t*-test. (*) P ≤ 0.05 is significant. Source data of **b** is provided as Supplementary Data 5. Raw data of **c** is given in Source Data File.

Supplementary Figure 5

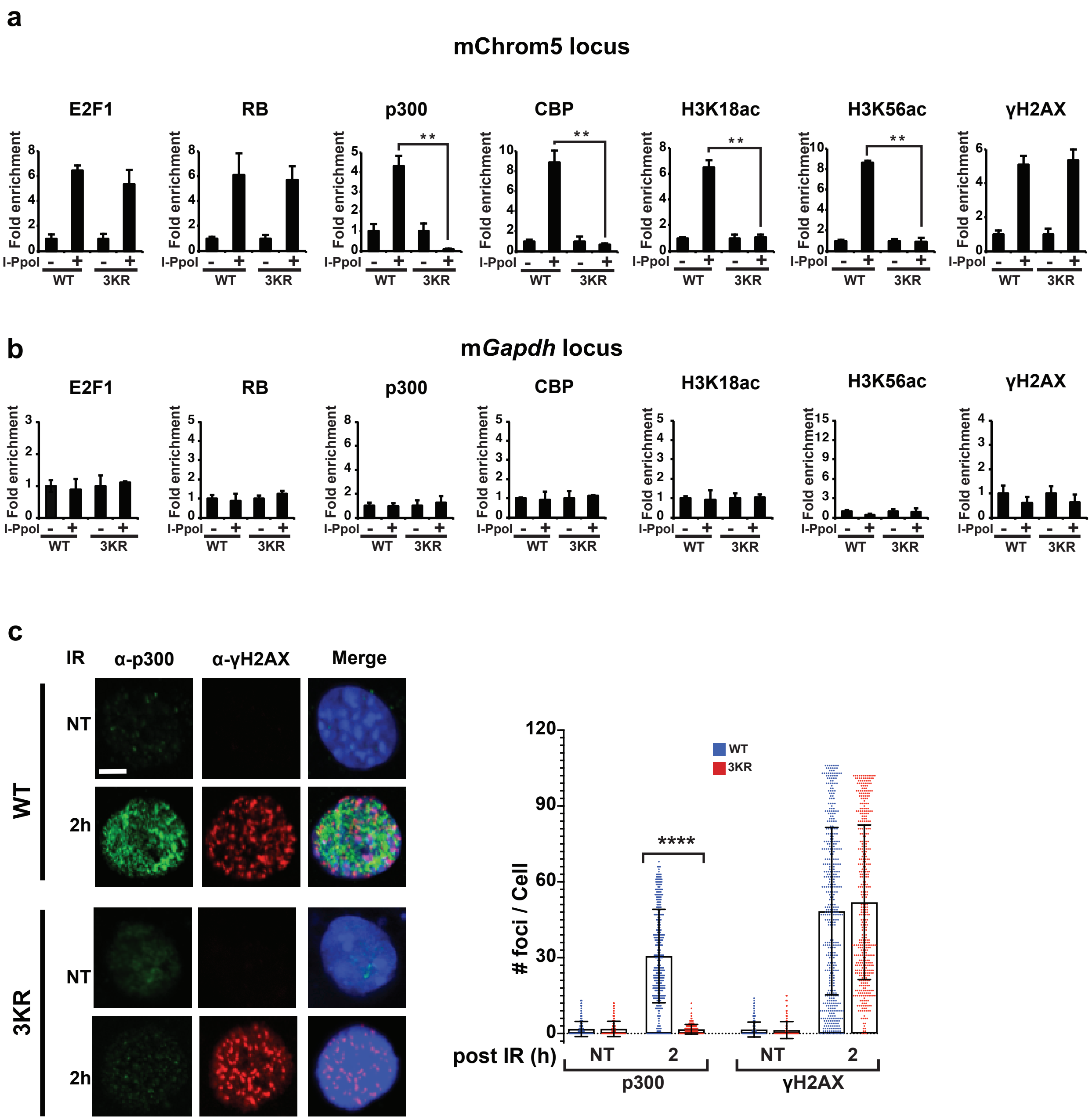

Supplementary Figure 5: E2F1 3KR mutation impairs recruitment of p300/CBP to DSBs. (a) The I-PpoI ChIP assay was performed using primary wild type (WT) and *E2f1*$^{3KR/3KR}$ (3KR) MEFs and occupancy of the indicated factors was determined for a region on mChrom5 locus. **(b)** The same assay was performed as above and occupancy was determined at the *Gapdh* locus. Graphs represent average ± SD of three independent experiments (n=3). P values were calculated by unpaired Student's *t*-test. (**) P ≤ 0.01 is highly significant. **(c)** Wild type and *E2f1*$^{3KR/3KR}$ MEFs were mock treated (NT) or exposed to 5 Gy of IR 2h prior to in situ extraction and fixation. Representative images show formation of IR-induced foci for p300 (green) and γH2AX (red) and merged with DAPI staining (blue) of nuclei. Bar, 10 μm. Quantification of p300 and γH2AX foci from the same experiment was performed. Each treatment group was in triplicate, and in total at least 450 cells (n > 450) were counted per treatment group. Graphs represent average ± SD of foci per cell. Overlaid scattered dot plot shows the distribution of foci. P values were calculated by unpaired Mann-Whitney *U* test. (****) P < 0.0001 is highly significant. Raw data of averages in graphs underlying **a** – **c** are given in Source Data File.

Supplementary Figure 6

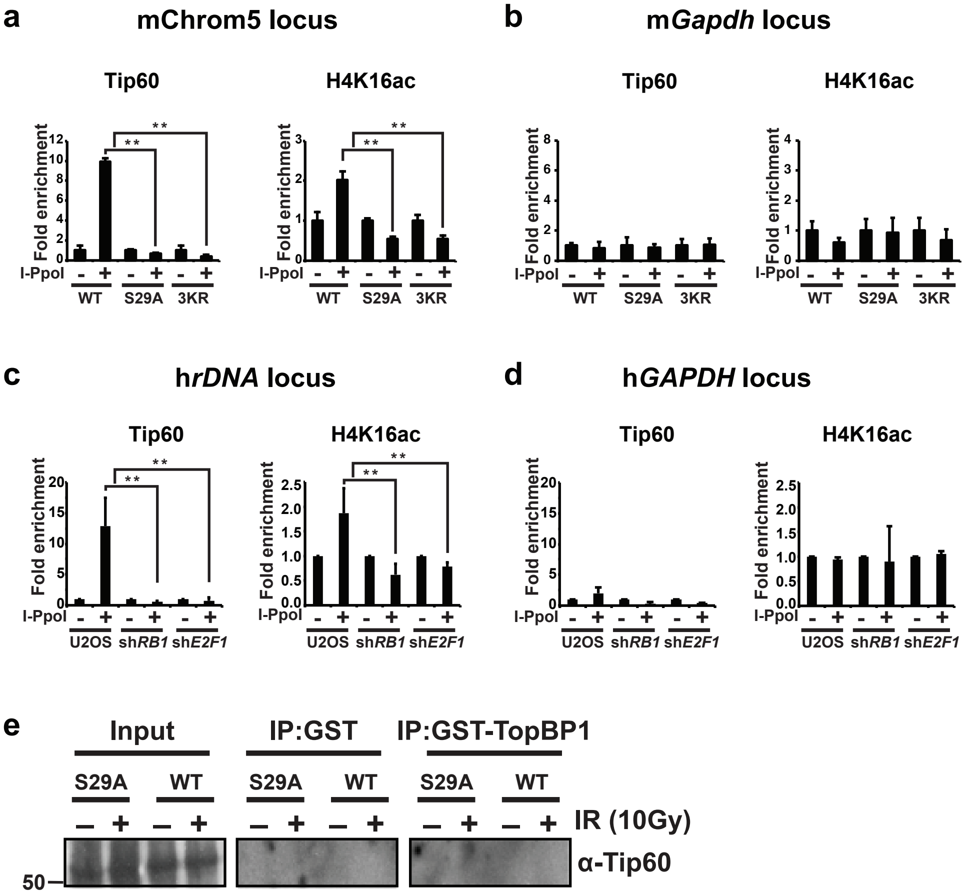

Supplementary Figure 6: Recruitment status of Tip60 and induction of H4K16ac at control and break sites. (a) The I-PpoI ChIP assay was performed using primary wild type (WT), *E2f1*$^{S29A/S29A}$ (S29A), and *E2f1*$^{3KR/3KR}$ (3KR) MEFs and occupancy of Tip60 and H4K16ac was determined for a region on mChrom5 locus. **(b)** The same assay was performed as above and occupancy was determined at the *Gapdh* locus. **(c and d)** The I-PpoI ChIP assay was performed using parental U2OS cells or U2OS cells depleted for RB (*shRB1*) or E2F1 (*shE2F1*) and occupancy of Tip60 and H4K16ac was determined at the *rDNA* locus **(c)** and *GAPDH* locus **(d)**. All experiments were done in triplicate (n=3). P values were calculated by unpaired Student's *t*-test. (**) P ≤ 0.01 is highly significant. **(e)** GST-TopBP1 (BRCT1-6) or GST control protein were incubated with whole cell extract from wild type (WT) or *E2f1*$^{S29A/S29A}$ (S29A) MEFs that were either untreated (-) or treated (+) with IR (10 Gy). Following pull down, binding of Tip60 was analyzed 2h post-IR by western blotting. From the same experiment CBP was also blotted which served as a positive control and is shown in Fig. 2a. Source data of **e** is provided as Supplementary Data 5. Raw data of **a** - **d** are in Source Data File.

Supplementary Figure 7

a

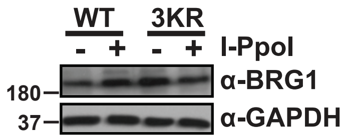

b

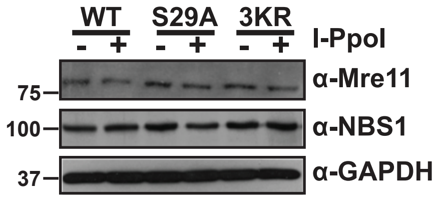

Supplementary Figure 7: The E2F1 knock-in mutations do not affect BRG1, Mre11 and NBS1 protein levels. (a) Western blot analysis for BRG1 and GAPDH was performed using whole cell extracts from primary wild type (WT) and *E2f1*$^{3KR/3KR}$ (3KR) MEFs without (-) or with (+) I-PpoI induction. (b) Western blot analysis for Mre11, NBS1 and GAPDH was performed using whole cell extracts from primary wild type (WT), *E2f1*$^{S29A/S29A}$ (S29A) and *E2f1*$^{3KR/3KR}$ (3KR) MEFs, plus (+) and minus (-) I-PpoI induction. Source data of **a** and **b** are provided as Supplementary Data 5.

Supplementary Table 1: List of reagents used in this study

REAGENT	SOURCE	IDENTIFIER
Dithiothreitol	Gold Biotechnology	27565-41-9
$^{15}NH_4Cl$	Sigma	299251
IPTG	Gold biotechnology	I2481C100
Glutatahione-Sepharose 4B beads	Thermo Fisher Sci	16101
NI-NTA Agarose	Qiagen	30250
PreScission protease	Home expressed	N/A
Streptavidin Agarose beads	EMD-Millipore	16-126
L-Glutathione Reduced	Sigma	G4251
I-CBP112	Xcess Biosciences Inc	M60128-2s
Formaldehyde	Sigma	F8775
4-hydroxy tamaoxifen	Sigma	H-7904
ChIP-Grade Protein G Magnetic Beads	Cell Signaling Technology	9006
Protease Inhibitor Cocktail tablets (Complete, EDTA-free)	Roche	11873580001
Phosphatase Inhibitor Cocktail tablets (PhosSTOP)	Roche	04906837001
Trichostatin A (TSA)	Sigma	T1952
EX-527	Selleckchem.com	S1541
iTaq Universal SYBR Green Supermix	Bio-Rad	172-5124
Western Lightning^{R} Plus - ECL	Perkin Elmer	NEL104001EA
DAPI	Invitrogen	D1306
Background Sniper	Biocare	BS966H
DaVinci Green	Biocare	PD900L
SuperScript II Reverse Transcriptase	Invitrogen	18064-014
dNTP Mix	Invitrogen	18427-013
Neocarzinostatin (NCS)	Sigma	N9162
Colcemid	Gibco	15212-012

Supplementary Table 2: List of antibodies used in this study

ANTIBODY	DILUTION	SOURCE	IDENTIFIER
anti-GST (Z-5)	WB – 1:1000	Santa Cruz Biotechnology	Cat#sc-459; RRID: AB_631586
anti-E2F1 (C-20) TransCruz	WB – 1:10000<br>ChIP – 6 μg	Santa Cruz Biotechnology	Cat#sc-193; RRID: AB_631394
anti-E2F1 (KH95)	WB – 1:1000	Santa Cruz Biotechnology	Cat#sc-251; RRID: AB_627476
Anti-Ac-lysine (AKL5C1)	WB – 1:1000	Santa Cruz Biotechnology	Cat#sc-32268; RRID: AB_627898
anti-RB (C-15) TransCruz	WB – 1:10000<br>ChIP – 6 μg	Santa Cruz Biotechnology	Cat#sc-50; RRID: AB_632339
anti-p300 (C-20) TransCruz	WB – 1:5000<br>ChIP – 6 μg<br>IF – 1:500	Santa Cruz Biotechnology	Cat#sc-585; RRID: AB_2231120
anti-CBP (A-22) TransCruz	WB – 1:5000<br>ChIP – 6 μg<br>IF – 1:250	Santa Cruz Biotechnology	Cat#sc-369; RRID: AB_631006
anti-H3K18ac	ChIP – 5 μl	EMD-Millipore	Cat#07-354; RRID: AB_441945
anti-H3K56ac	ChIP – 10 μl	Cell Signaling Technology	Cat#4243; RRID: N/A
anti-γH2AX (Ser139)	WB – 1:2000<br>ChIP – 6 μg	Cell Signaling Technology	Cat#2577; RRID: AB_2118010
anti-γH2AX (Ser139)	IF – 1:10000	EMD-Millipore	Cat#05-636; RRID: AB_309864
anti-pATM (Ser1981)	WB – 1:2000	Cell Signaling Technology	Cat#5883; RRID: AB_10835213
anti-Cleaved Lamin A	IHC – 1:100	Cell Signaling Technology	Cat#2035; RRID: N/A
anti-53BP1	IF – 1:250	Bethyl Laboratories, Inc.	Cat#A300-272A; RRID: AB_185520
anti-GAPDH (clone 6C5)	WB – 1:10000	EMD-Millipore	Cat#MAB374; RRID: AB_2107445
anti-Tip60	WB – 1:1000<br>ChIP – 6 μg	Abcam	Cat#ab23886; RRID: AB_778485
anti-H4K16ac	ChIP – 6 μg	EMD-Millipore	Cat#07-329; RRID: AB_310525
anti-BRG1 (H-88)	WB – 1:1000<br>ChIP – 6 μg	Santa Cruz Biotechnology	Cat#sc-10768; RRID: AB_2255022
anti-Histone H3 (D1H2) XP	WB – 1:5000<br>ChIP – 10 μl	Cell Signaling Technology	Cat#4499; RRID: AB_10544537
anti-NBS1	WB – 1:1000<br>ChIP – 10 μl	Cell Signaling Technology	Cat#3002; RRID: AB_331499
anti-Mre11	WB – 1:4000<br>ChIP – 10 μl	Cell Signaling Technology	Cat#4895; RRID: AB_2145100
anti-phospho p53 (Ser15)	WB – 1:2000	Cell Signaling Technology	Cat#9284; RRID: AB_331464

Cat# - Catalog Number; RRID – Research Resource Identifiers

WB – Western blotting; ChIP – Chromatin Immunoprecipitation; IF – Immunofluorescence; IHC - Immunohistochemistry

Supplementary Table 3: List of primers used in this study

PRIMER	SOURCE	IDENTIFIER
Random Hexamer<br>5'-d (NNNNNN) -3' N = G, A, T or C	Thermo Scientific	SO142
mChrom5 locus_Forward:<br>TGGGAATCTCATTCATCCATT	This paper	N/A
mChrom5 locus_Reverse:<br>CCAGAAGGTCAGAAGGATCG	This paper	N/A
mChrom10 locus_Forward:<br>CATGCATGAATGGAATGAGGA	This paper	N/A
mChrom10 locus_Reverse:<br>CAGGGAAGGAAGAGCATGAG	This paper	N/A
m*Gapdh* locus_Forward:<br>TTCTCGGGCAAAAATGAGAG	This paper	N/A
m*Gapdh* locus_Reverse:<br>TTCCATCCTCCAGAAACCAG	This paper	N/A
hChrom1 locus:<br>TGCTGCTTTTTCTTCTTCTCC<br>CTTCTTTCCCACCAAGTCTTC	[22]Berkovich et al., 2008	N/A
h*rDNA* locus:<br>TGGAGCAGAAGGGCAAAAGC<br>TAGGAAGAGCCGACATCGAAGG	[22]Berkovich et al., 2008	N/A
h*GAPDH* locus:<br>TCGGTTCTTGCCTCTTGTC<br>CTTCCATTCTGTCTTCCACTC	[22]Berkovich et al., 2008	N/A
p73 mRNA_Forward:<br>GCACCTACTTTGACCTCCCC	PrimerBank	30794514a1
p73 mRNA_Reverse:<br>GCACTGCTGAGCAAATTGAAC	PrimerBank	30794514a1
Apaf1 mRNA_Forward:<br>AGTAATGGGTCCTAAGCATGTTG	PrimerBank	6857755a1
Apaf1 mRNA_Reverse:<br>GCGATTGGGAAAATCACGTAAAA	PrimerBank	6857755a1
Casp3 mRNA_Forward:<br>TGGTGATGAAGGGGTCATTTATG	PrimerBank	24416451a1
Casp3 mRNA_Reverse:<br>TTCGGCTTTCCAGTCAGACTC	PrimerBank	24416451a1
Gapdh mRNA_Forward:<br>AGAACATCATCCCTGCATCC	This paper	N/A
Gapdh mRNA_Reverse:<br>CACATTGGGGGTAGGAACAC	This paper	N/A
