# Supplementary material for: E2F1 acetylation directs p300/CBP-mediated histone acetylation at DNA double-strand breaks to facilitate repair
Source: Nat Commun. 2019 Oct 30;10:4951. doi: 10.1038/s41467-019-12861-8 (PMC6821830; doi:10.1038/s41467-019-12861-8)
Supplement: Supplementary file 3 — Reporting Summary [file 41467_2019_12861_MOESM3_ESM.pdf]

nature research

Corresponding author(s): David G Johnson

Last updated by author(s): Sep 25, 2019

Reporting Summary

Nature Research wishes to improve the reproducibility of the work that we publish. This form provides structure for consistency and transparency in reporting. For further information on Nature Research policies, see Authors & Referees and the Editorial Policy Checklist.

Statistics

For all statistical analyses, confirm that the following items are present in the figure legend, table legend, main text, or Methods section.

n/a	Confirmed	
☐	☒	The exact sample size (n) for each experimental group/condition, given as a discrete number and unit of measurement
☐	☒	A statement on whether measurements were taken from distinct samples or whether the same sample was measured repeatedly
☐	☒	The statistical test(s) used AND whether they are one- or two-sided<br>*Only common tests should be described solely by name; describe more complex techniques in the Methods section.*
☒	☐	A description of all covariates tested
☒	☐	A description of any assumptions or corrections, such as tests of normality and adjustment for multiple comparisons
☐	☒	A full description of the statistical parameters including central tendency (e.g. means) or other basic estimates (e.g. regression coefficient) AND variation (e.g. standard deviation) or associated estimates of uncertainty (e.g. confidence intervals)
☐	☒	For null hypothesis testing, the test statistic (e.g. F, t, r) with confidence intervals, effect sizes, degrees of freedom and P value noted<br>*Give P values as exact values whenever suitable.*
☒	☐	For Bayesian analysis, information on the choice of priors and Markov chain Monte Carlo settings
☒	☐	For hierarchical and complex designs, identification of the appropriate level for tests and full reporting of outcomes
☒	☐	Estimates of effect sizes (e.g. Cohen's d, Pearson's r), indicating how they were calculated

Our web collection on statistics for biologists contains articles on many of the points above.

Software and code

Policy information about availability of computer code

Data collection	Information of all the commercial devises used for data collection is given in Methods section
Data analysis	Data in this manuscript are generated using commonly available commercial software and algorithms and are detailed in the corresponding methods section. Specific computer code is not applicable.

For manuscripts utilizing custom algorithms or software that are central to the research but not yet described in published literature, software must be made available to editors/reviewers. We strongly encourage code deposition in a community repository (e.g. GitHub). See the Nature Research guidelines for submitting code & software for further information.

Data

Policy information about availability of data

All manuscripts must include a data availability statement. This statement should provide the following information, where applicable:

- Accession codes, unique identifiers, or web links for publicly available datasets
- A list of figures that have associated raw data
- A description of any restrictions on data availability

Knock-in mouse models used in this study are registered in Mouse Genome Informatics (MGI) database. Strain details of ‘E2f1S29A/S29A (FVB.Cg-E2f1tm1.1Dgj) can be found in MGI ID:5755411 [http://www.informatics.jax.org/allele/MGI:5637520]’, and ‘E2f13KR/3KR (E2f1em1Dgj) in MGI ID:6313577 [http://www.informatics.jax.org/allele/key/882523]’. RNA-seq data are available in Gene Expression Omnibus (GEO) repository under accession ‘GSE135360 [https://www.ncbi.nlm.nih.gov/geo/query/acc.cgi?acc=GSE135360]’. All data supporting the findings of this study are available within the article and its supplementary information files. Additional information and relevant data will be available from the corresponding author upon reasonable request. Supplementary Data 5 contains raw data of blots/gels underlying Fig. 1b, c; 2a, b; 4b, c; 5b; 6b, d; and Supplementary Figures 1c; 2a; 3d; 4b; 6e; 7a, b. Source Data file contains raw data of all reported averages in graphs and charts underlying Main Figures, Fig. 3a - c; Fig. 4a, d and e; Fig. 5a, d – f; Fig. 6a and c; Fig. 7b – e; and Supplementary Figures 2b and c; 3b, c and e; 4c; 5a – c and 6a – d.

Field-specific reporting

Please select the one below that is the best fit for your research. If you are not sure, read the appropriate sections before making your selection.

☒ Life sciences ☐ Behavioural & social sciences ☐ Ecological, evolutionary & environmental sciences

For a reference copy of the document with all sections, see nature.com/documents/nr-reporting-summary-flat.pdf

Life sciences study design

All studies must disclose on these points even when the disclosure is negative.

Sample size	Sample size was based on traditional experimental approach in molecular and cell biology. In general, the sample sizes for mice studies, minimum 3 mice per genotype per treatment conditions were used. For quantitative experiments, like qPCR, samples were prepared in triplicates.
Data exclusions	No data were excluded.
Replication	In Figure legends, number of biological replications was stated for each experiment.
Randomization	The experiments did not require sample randomization. Samples were handled the same way in all experiments.
Blinding	The investigators were not blinded during data collection or outcome assessment. This approach is considered standard for biochemical experiments performed in this study.

Reporting for specific materials, systems and methods

We require information from authors about some types of materials, experimental systems and methods used in many studies. Here, indicate whether each material, system or method listed is relevant to your study. If you are not sure if a list item applies to your research, read the appropriate section before selecting a response.

Materials & experimental systems

n/a	Involved in the study	
☐	☒	Antibodies
☐	☒	Eukaryotic cell lines
☒	☐	Palaeontology
☐	☒	Animals and other organisms
☒	☐	Human research participants
☒	☐	Clinical data

Methods

n/a	Involved in the study	
☒	☐	ChIP-seq
☒	☐	Flow cytometry
☒	☐	MRI-based neuroimaging

Antibodies

Antibodies used	All antibodies used in this study are listed in Supplementary Table 2 with their source and catalog number
Validation	Antibodies suitable for specific purposes were purchased and the validation was performed by the vendors. For more details, each antibody used in this study is provided with Research Resource Identifiers (RRID) in Supplementary Table 2.

Eukaryotic cell lines

Policy information about cell lines

Cell line source(s)	The following cell lines were used in this study: Primary mouse embryonic fibroblasts (MEFs) isolated from wild type, E2f1S29A/S29A and E2f13KR/3KR knock-in mice; U2OS parental and shRNA-expressing cell lines shRB1 and shE2F1.
Authentication	Primary MEFs are isolated from crossing heterozygous or homozygous mice of each strain following standard procedures. U2OS cells are from ATCC. shRNA-expressing U2OS derivatives, shRB1 and shE2F1 are published (Velez-Cruz et al, 2016).
Mycoplasma contamination	Primary MEFs do not require assessment of mycoplasma contamination. U2OS cells were assessed by ATCC.
Commonly misidentified lines (See ICLAC register)	Not applicable.

Animals and other organisms

Policy information about studies involving animals; ARRIVE guidelines recommended for reporting animal research

Laboratory animals	Wild type, E2f1S29A/S29A and E2f13KR/3KR knock-in mice of FVB strains are used in this study. The wild type FVB mice (3 to 4 weeks old) are purchased (Harlan Laboratories, IN) and the knock-in lines are produced in house. Mice of both sex were randomly allocated to experiments at the age of 6 to 8 weeks. A detailed description of maintenance of mice is given in Methods section.
Wild animals	Not applicable.
Field-collected samples	Not applicable.
Ethics oversight	All animal experiments complied with the National Research Council's guide for the Care and Use of Laboratory Animals and were approved by the University of Texas MD Anderson Institutional Animal Care and Use Committee (IACUC).

Note that full information on the approval of the study protocol must also be provided in the manuscript.
