# Supplementary material for: E2F1 acetylation directs p300/CBP-mediated histone acetylation at DNA double-strand breaks to facilitate repair
Source: Nat Commun. 2019 Oct 30;10:4951. doi: 10.1038/s41467-019-12861-8 (PMC6821830; doi:10.1038/s41467-019-12861-8)
Supplement: Supplementary file 4 — Description of Additional Supplementary Files [file 41467_2019_12861_MOESM4_ESM.pdf]

Description of Additional Supplementary Files

File Name: Supplementary Data 1
Description: Genes enriched in wild type compared 3KR, untreated

File Name: Supplementary Data 2
Description: Genes enriched in wild type compared 3KR, NCS treated

File Name: Supplementary Data 3
Description: Genes enriched in 3KR compared wild type, untreated

File Name: Supplementary Data 4
Description: Genes enriched in 3KR compared wild type, NCS treated

File Name: Supplementary Data 5
Description: Uncropped Images of blots/gels.
