# Supplementary material for: E2F1 acetylation directs p300/CBP-mediated histone acetylation at DNA double-strand breaks to facilitate repair
Source: Nat Commun. 2019 Oct 30;10:4951. doi: 10.1038/s41467-019-12861-8 (PMC6821830; doi:10.1038/s41467-019-12861-8)
Supplement: Supplementary file 5 — Supplementary Data 1 [file 41467_2019_12861_MOESM5_ESM.pdf]

Supplementary Data 1: List of gene sets significantly enriched in wild type MEFs compared to *E2f1*$^{3KR/3KR}$ before DNA damage. False Discovery Rate (FDR), q value ≤ 0.05.

#	NAME	FDR q-value
1	NEUROTRANSMITTER_TRANSPORT(4)	0.002
2	NEUROTRANSMITTER_SECRETION(5)	0.001
3	REGULATION_OF_AXON_EXTENSION(5)	0.003
4	REGULATION_OF_NEUROTRANSMITTER_LEVELS(4)	0.003
5	REGULATION_OF_EXTENT_OF_CELL_GROWTH(5)	0.005
6	POSITIVE_REGULATION_OF_NEURON_DIFFERENTIATION(5)	0.010
7	REGULATION_OF_POSTSYNAPTIC_MEMBRANE_POTENTIAL(4)	0.013
8	AUTONOMIC_NERVOUS_SYSTEM_DEVELOPMENT(5)	0.012
9	POSITIVE_REGULATION_OF_AXON_EXTENSION(5)	0.012
10	ADULT_LOCOMOTORY_BEHAVIOR(4)	0.011
11	POSITIVE_REGULATION_OF_CELL_MORPHOGENESIS_INVOLVED_IN_DIFFERENTIATION(4)	0.010
12	FILOPODIUM_ASSEMBLY(6)	0.013
13	GLIOGENESIS(7)	0.014
14	PARASYMPATHETIC_NERVOUS_SYSTEM_DEVELOPMENT(5)	0.013
15	CELL_CELL_ADHESION(4)	0.012
16	CELL_CELL_ADHESION_VIA_PLASMA_MEMBRANE_ADHESION_MOLECULES(5)	0.014
17	NEUROBLAST_PROLIFERATION(5)	0.013
18	REGULATION_OF_NEUROBLAST_PROLIFERATION(6)	0.017
19	REGULATION_OF_MEMBRANE_POTENTIAL(4)	0.017
20	GLIAL_CELL_DIFFERENTIATION(6)	0.016
21	NERVE_DEVELOPMENT(4)	0.017
22	LENS_MORPHOGENESIS_IN_CAMERA_TYPE_EYE(4)	0.018
23	REGULATION_OF_EXCITATORY_POSTSYNAPTIC_MEMBRANE_POTENTIAL(5)	0.018
24	PREGANGLIONIC_PARASYMPATHETIC_FIBER_DEVELOPMENT(5)	0.020
25	REGULATION_OF_CELL_SIZE(5)	0.020
26	POSITIVE_REGULATION_OF_NERVOUS_SYSTEM_DEVELOPMENT(4)	0.020
27	ADULT_WALKING_BEHAVIOR(5)	0.020
28	POSITIVE_REGULATION_OF_AXONOGENESIS(5)	0.020
29	POSITIVE_REGULATION_OF_NEURON_PROJECTION_DEVELOPMENT(5)	0.020
30	HOMOPHILIC_CELL_ADHESION_VIA_PLASMA_MEMBRANE_ADHESION_MOLECULES(6)	0.022
31	POSITIVE_REGULATION_OF_NEUROGENESIS(5)	0.022
32	BLOOD_VESSEL_REMODELING(5)	0.024
33	REGULATION_OF_AXONOGENESIS(6)	0.024
34	AXON_EXTENSION(6)	0.023
35	POSITIVE_REGULATION_OF_CELL_PROJECTION_ORGANIZATION(4)	0.023
36	THYROID_GLAND_DEVELOPMENT(5)	0.024
37	NEURON_PROJECTION_EXTENSION(5)	0.025
38	LOCOMOTORY_BEHAVIOR(3)	0.027
39	REGULATION_OF_NEURAL_PRECURSOR_CELL_PROLIFERATION(5)	0.028
40	FOREBRAIN_NEURON_DIFFERENTIATION(6)	0.028
41	POSITIVE_REGULATION_OF_NEURAL_PRECURSOR_CELL_PROLIFERATION(5)	0.028
42	REGULATION_OF_NEUROTRANSMITTER_SECRETION(4)	0.031
43	OLIGODENDROCYTE_DIFFERENTIATION(6)	0.034
44	DORSAL_SPINAL_CORD_DEVELOPMENT(4)	0.033
45	DEVELOPMENTAL_CELL_GROWTH(4)	0.033
46	POSITIVE_REGULATION_OF_NEUROBLAST_PROLIFERATION(6)	0.033
47	NEGATIVE_REGULATION_OF_NEURAL_PRECURSOR_CELL_PROLIFERATION(5)	0.039

48	POSITIVE_REGULATION_OF_CELL_DEVELOPMENT(5)	0.039
49	AXON_DEVELOPMENT(6)	0.039
50	CELL_MATURATION(5)	0.039
51	LENS_DEVELOPMENT_IN_CAMERA_TYPE_EYE(4)	0.040
52	MEMBRANE_HYPERPOLARIZATION(5)	0.043
53	REGULATION_OF_CELL_PROJECTION_ORGANIZATION(4)	0.049
54	SIGNAL_RELEASE(5)	0.050
55	REGULATION_OF_NEURON_DIFFERENTIATION(7)	0.049
