# Supplementary material for: E2F1 acetylation directs p300/CBP-mediated histone acetylation at DNA double-strand breaks to facilitate repair
Source: Nat Commun. 2019 Oct 30;10:4951. doi: 10.1038/s41467-019-12861-8 (PMC6821830; doi:10.1038/s41467-019-12861-8)
Supplement: Supplementary file 6 — Supplementary Data 2 [file 41467_2019_12861_MOESM6_ESM.pdf]

**Supplementary Data 2: List of gene sets significantly enriched in wild type MEFs compared to *E2f1*$^{3KR/3KR}$ after DNA damage. False Discovery Rate (FDR), q value ≤ 0.05.**

| # | NAME | FDR q-value |
|---|---|---|
| 1 | CELL_CELL_ADHESION_VIA_PLASMA_MEMBRANE_ADHESION_MOLECULES(5) | 0.001 |
| 2 | CELL_CELL_ADHESION(4) | 0.000 |
| 3 | NEUROTRANSMITTER_TRANSPORT(4) | 0.001 |
| 4 | GLIAL_CELL_DIFFERENTIATION(6) | 0.001 |
| 5 | POSITIVE_REGULATION_OF_NEURON_DIFFERENTIATION(5) | 0.002 |
| 6 | NEUROTRANSMITTER_SECRETION(5) | 0.002 |
| 7 | POSITIVE_REGULATION_OF_NEURON_PROJECTION_DEVELOPMENT(5) | 0.001 |
| 8 | GLIOGENESIS(7) | 0.001 |
| 9 | POSITIVE_REGULATION_OF_NERVOUS_SYSTEM_DEVELOPMENT(4) | 0.002 |
| 10 | HOMOPHILIC_CELL_ADHESION_VIA_PLASMA_MEMBRANE_ADHESION_MOLECULES(6) | 0.002 |
| 11 | REGULATION_OF_NEUROTRANSMITTER_LEVELS(4) | 0.002 |
| 12 | REGULATION_OF_AXON_EXTENSION(5) | 0.002 |
| 13 | AUTONOMIC_NERVOUS_SYSTEM_DEVELOPMENT(5) | 0.002 |
| 14 | REGULATION_OF_EXTENT_OF_CELL_GROWTH(5) | 0.002 |
| 15 | ADULT_LOCOMOTORY_BEHAVIOR(4) | 0.003 |
| 16 | REGULATION_OF_POSTSYNAPTIC_MEMBRANE_POTENTIAL(4) | 0.004 |
| 17 | REGULATION_OF_MEMBRANE_POTENTIAL(4) | 0.004 |
| 18 | OLIGODENDROCYTE_DIFFERENTIATION(6) | 0.005 |
| 19 | REGULATION_OF_NEUROBLAST_PROLIFERATION(6) | 0.005 |
| 20 | POSITIVE_REGULATION_OF_AXONOGENESIS(5) | 0.005 |
| 21 | BLOOD_VESSEL_REMODELING(5) | 0.005 |
| 22 | POSITIVE_REGULATION_OF_CELL_MORPHOGENESIS_INVOLVED_IN_DIFFERENTIATION(4) | 0.005 |
| 23 | POSITIVE_REGULATION_OF_NEUROGENESIS(5) | 0.005 |
| 24 | AXON_DEVELOPMENT(6) | 0.005 |
| 25 | POSITIVE_REGULATION_OF_NEUROBLAST_PROLIFERATION(6) | 0.005 |
| 26 | HETEROPHILIC_CELL_CELL_ADHESION_VIA_PLASMA_MEMBRANE_CELL_ADHESION_MOLECULES(6) | 0.006 |
| 27 | LOCOMOTORY_BEHAVIOR(3) | 0.007 |
| 28 | REGULATION_OF_CELL_SIZE(5) | 0.007 |
| 29 | REGULATION_OF_AXONOGENESIS(6) | 0.006 |
| 30 | POSITIVE_REGULATION_OF_NEURAL_PRECURSOR_CELL_PROLIFERATION(5) | 0.006 |
| 31 | POSITIVE_REGULATION_OF_CELL_PROJECTION_ORGANIZATION(4) | 0.007 |
| 32 | POSITIVE_REGULATION_OF_AXON_EXTENSION(5) | 0.006 |
| 33 | REGULATION_OF_NEURON_PROJECTION_DEVELOPMENT(5) | 0.008 |
| 34 | REGULATION_OF_NEURAL_PRECURSOR_CELL_PROLIFERATION(5) | 0.009 |
| 35 | NEURON_PROJECTION_DEVELOPMENT(5) | 0.010 |
| 36 | REGULATION_OF_NEURON_DIFFERENTIATION(7) | 0.011 |
| 37 | INTERMEDIATE_FILAMENT_CYTOSKELETON_ORGANIZATION(5) | 0.011 |
| 38 | INTERMEDIATE_FILAMENT_BASED_PROCESS(4) | 0.011 |
| 39 | AXONOGENESIS(7) | 0.011 |
| 40 | LENS_MORPHOGENESIS_IN_CAMERA_TYPE_EYE(4) | 0.010 |
| 41 | BEHAVIORAL_DEFENSE_RESPONSE(3)&BEHAVIORAL_FEAR_RESPONSE(4) | 0.011 |
| 42 | REGULATION_OF_EXCITATORY_POSTSYNAPTIC_MEMBRANE_POTENTIAL(5) | 0.011 |
| 43 | REGULATION_OF_CELL_PROJECTION_ORGANIZATION(4) | 0.012 |
| 44 | SODIUM_ION_TRANSMEMBRANE_TRANSPORT(6) | 0.012 |
| 45 | REGULATION_OF_NERVOUS_SYSTEM_DEVELOPMENT(5) | 0.015 |
| 46 | DEVELOPMENTAL_CELL_GROWTH(4) | 0.016 |
| 47 | REGULATION_OF_NEUROGENESIS(6) | 0.016 |

| | | |
|---|---|---|
| 48 | NEURON_NEURON_SYNAPTIC_TRANSMISSION(4) | 0.017 |
| 49 | POSITIVE_REGULATION_OF_CELL_DEVELOPMENT(5) | 0.018 |
| 50 | NEURON_PROJECTION_MORPHOGENESIS(6) | 0.019 |
| 51 | AXON_EXTENSION(6) | 0.021 |
| 52 | REGULATION_OF_CELL_MORPHOGENESIS_INVOLVED_IN_DIFFERENTIATION(5) | 0.021 |
| 53 | NEURON_MATURATION(6) | 0.022 |
| 54 | MULTICELLULAR_ORGANISMAL_SIGNALING(4) | 0.026 |
| 55 | DEVELOPMENTAL_MATURATION(4) | 0.026 |
| 56 | BLOOD_CIRCULATION(6) | 0.028 |
| 57 | REGULATION_OF_CELL_DEVELOPMENT(5) | 0.029 |
| 58 | PARASYMPATHETIC_NERVOUS_SYSTEM_DEVELOPMENT(5) | 0.029 |
| 59 | SYNAPSE_ORGANIZATION(4) | 0.028 |
| 60 | CELL_MORPHOGENESIS_INVOLVED_IN_NEURON_DIFFERENTIATION(6) | 0.029 |
| 61 | REGULATION_OF_CELL_MORPHOGENESIS(4) | 0.028 |
| 62 | NEUROBLAST_PROLIFERATION(5) | 0.030 |
| 63 | CELL_PART_MORPHOGENESIS(5) | 0.030 |
| 64 | CELL_PROJECTION_MORPHOGENESIS(5) | 0.030 |
| 65 | NEUROLOGICAL_SYSTEM_PROCESS(5) | 0.030 |
| 66 | SCHWANN_CELL_DIFFERENTIATION(6) | 0.034 |
| 67 | NERVE_DEVELOPMENT(4) | 0.034 |
| 68 | CELL_MATURATION(5) | 0.035 |
| 69 | EPITHELIAL_TUBE_BRANCHING_INVOLVED_IN_LUNG_MORPHOGENESIS(6) | 0.040 |
| 70 | NEURON_PROJECTION_EXTENSION(5) | 0.040 |
| 71 | SENSORY_PERCEPTION(6) | 0.041 |
| 72 | NEURAL_CREST_CELL_DEVELOPMENT(7) | 0.042 |
| 73 | NEGATIVE_REGULATION_OF_CELL_DEVELOPMENT(5) | 0.041 |
| 74 | REGULATION_OF_NEURON_DEATH(5) | 0.041 |
| 75 | LENS_DEVELOPMENT_IN_CAMERA_TYPE_EYE(4) | 0.045 |
| 76 | FILOPODIUM_ASSEMBLY(6) | 0.044 |
| 77 | THYROID_GLAND_DEVELOPMENT(5) | 0.044 |
| 78 | RESPIRATORY_SYSTEM_PROCESS(5) | 0.043 |
| 79 | NEURON_PROJECTION_GUIDANCE(5)&AXON_GUIDANCE(6) | 0.043 |
| 80 | ADULT_WALKING_BEHAVIOR(5) | 0.043 |
| 81 | DETECTION_OF_MECHANICAL_STIMULUS_INVOLVED_IN_SENSORY_PERCEPTION(5) | 0.042 |
| 82 | NEURON_DEATH(5) | 0.045 |
| 83 | CELLULAR_POTASSIUM_ION_TRANSPORT(4) | 0.045 |
| 84 | ADULT_BEHAVIOR(4) | 0.045 |
| 85 | LUNG_EPITHELIUM_DEVELOPMENT(5) | 0.045 |
| 86 | TRANSMISSION_OF_NERVE_IMPULSE(5) | 0.046 |
| 87 | CELL_CELL_SIGNALING(4) | 0.045 |
| 88 | NEGATIVE_REGULATION_OF_NEURON_DEATH(5) | 0.045 |
| 89 | AMEBOIDAL_TYPE_CELL_MIGRATION(5) | 0.045 |
| 90 | NEGATIVE_REGULATION_OF_NEUROGENESIS(5) | 0.045 |
| 91 | CIRCULATORY_SYSTEM_PROCESS(5) | 0.045 |
| 92 | FEAR_RESPONSE(5) | 0.045 |
| 93 | NEGATIVE_REGULATION_OF_NEURAL_PRECURSOR_CELL_PROLIFERATION(5) | 0.049 |
| 94 | REGULATION_OF_NEURON_APOPTOTIC_PROCESS(6) | 0.049 |
| 95 | GLIAL_CELL_DEVELOPMENT(5) | 0.050 |
| 96 | DEVELOPMENTAL_PIGMENTATION(4) | 0.050 |
