# Supplementary material for: E2F1 acetylation directs p300/CBP-mediated histone acetylation at DNA double-strand breaks to facilitate repair
Source: Nat Commun. 2019 Oct 30;10:4951. doi: 10.1038/s41467-019-12861-8 (PMC6821830; doi:10.1038/s41467-019-12861-8)
Supplement: Supplementary file 7 — Supplementary Data 3 [file 41467_2019_12861_MOESM7_ESM.pdf]

Supplementary Data 3: List of gene sets significantly enriched in *E2f1*$^{3KR/3KR}$ compared to wild type MEFs before DNA damage. False Discovery Rate (FDR), q value ≤ 0.05.

**#**	**NAME**	**FDR q-value**
1	LEUKOCYTE_CHEMOTAXIS(4)	0.000
2	CELL_CHEMOTAXIS(5)	0.000
3	RESPONSE_TO_OTHER_ORGANISM(3)&RESPONSE_TO_EXTERNAL_BIOTIC_STIMULUS(4)	0.000
4	RESPONSE_TO_BIOTIC_STIMULUS(3)	0.000
5	RESPONSE_TO_BACTERIUM(4)	0.000
6	GRANULOCYTE_MIGRATION(5)	0.000
7	RESPONSE_TO_CYTOKINE(5)	0.000
8	DEFENSE_RESPONSE_TO_OTHER_ORGANISM(4)	0.000
9	MYELOID_LEUKOCYTE_MIGRATION(4)	0.000
10	NEUTROPHIL_CHEMOTAXIS(6)	0.000
11	GRANULOCYTE_CHEMOTAXIS(5)	0.000
12	NEUTROPHIL_MIGRATION(6)	0.000
13	DEFENSE_RESPONSE_TO_BACTERIUM(5)	0.000
14	CELLULAR_RESPONSE_TO_CYTOKINE_STIMULUS(6)	0.000
15	RESPONSE_TO_VIRUS(4)	0.000
16	INTERSPECIES_INTERACTION_BETWEEN_ORGANISMS(3)&SYMBIOSIS_ENCOMPASSING_MUTUALISM_THROUGH_PARASITISM(4)	0.000
17	INNATE_IMMUNE_RESPONSE(4)	0.000
18	RESPONSE_TO_INTERFERON_BETA(6)	0.000
19	CELLULAR_RESPONSE_TO_INTERFERON_BETA(7)	0.000
20	REGULATION_OF_RESPONSE_TO_BIOTIC_STIMULUS(4)	0.000
21	POSITIVE_REGULATION_OF_PHAGOCYTOSIS(5)	0.000
22	REGULATION_OF_MULTI_ORGANISM_PROCESS(3)	0.000
23	ANTIGEN_PROCESSING_AND_PRESENTATION(3)	0.000
24	LEUKOCYTE_MIGRATION(3)	0.000
25	REGULATION_OF_PHAGOCYTOSIS(5)	0.000
26	POSITIVE_REGULATION_OF_LEUKOCYTE_CHEMOTAXIS(5)	0.000
27	POSITIVE_REGULATION_OF_RESPONSE_TO_EXTERNAL_STIMULUS(4)	0.000
28	IMMUNE_RESPONSE(3)	0.001
29	IMMUNE_EFFECTOR_PROCESS(3)	0.001
30	CYTOKINE_PRODUCTION(4)	0.001
31	POSITIVE_REGULATION_OF_DEFENSE_RESPONSE(4)	0.001
32	DEFENSE_RESPONSE(4)	0.001
33	REGULATION_OF_SYMBIOSIS_ENCOMPASSING_MUTUALISM_THROUGH_PARASITISM(4)	0.001
34	TYPE_I_INTERFERON_PRODUCTION(5)	0.001
35	POSITIVE_REGULATION_OF_CYTOKINE_SECRETION(5)	0.001
36	POSITIVE_REGULATION_OF_CYTOKINE_PRODUCTION(4)	0.001
37	ACTIVATION_OF_INNATE_IMMUNE_RESPONSE(4)	0.001
38	RESPONSE_TO_MOLECULE_OF_BACTERIAL_ORIGIN(5)	0.001
39	REGULATION_OF_B_CELL_DIFFERENTIATION(7)	0.001
40	CYTOKINE_SECRETION(5)	0.001
41	POSITIVE_REGULATION_OF_LEUKOCYTE_MIGRATION(4)	0.001
42	POSITIVE_REGULATION_OF_IMMUNE_SYSTEM_PROCESS(3)	0.001
43	REGULATION_OF_INNATE_IMMUNE_RESPONSE(5)	0.001
44	REGULATION_OF_DEFENSE_RESPONSE(5)	0.001
45	LEUKOCYTE_MEDIATED_IMMUNITY(4)	0.002
46	NEGATIVE_REGULATION_OF_MULTI_ORGANISM_PROCESS(3)	0.002

47	PHAGOCYTOSIS_ENGULFMENT(5)	0.002
48	REGULATION_OF_CYTOKINE_PRODUCTION(4)	0.002
49	DEFENSE_RESPONSE_TO_GRAM_POSITIVE_BACTERIUM(6)	0.002
50	HEART_TRABECULA_MORPHOGENESIS(5)	0.002
51	MALE_SEX_DIFFERENTIATION(5)	0.002
52	DEFENSE_RESPONSE_TO_PROTOZOAN(5)	0.002
53	REGULATION_OF_IMMUNE_SYSTEM_PROCESS(3)	0.002
54	RESPONSE_TO_LIPOPOLYSACCHARIDE(5)	0.002
55	ANTIGEN_PROCESSING_AND_PRESENTATION_OF_PEPTIDE_ANTIGEN(4)	0.002
56	MYELOID_DENDRITIC_CELL_ACTIVATION(5)	0.003
57	PHAGOCYTOSIS(4)	0.003
58	REGULATION_OF_LEUKOCYTE_CHEMOTAXIS(5)	0.003
59	DEFENSE_RESPONSE_TO_VIRUS(4)	0.003
60	DEFENSE_RESPONSE_TO_GRAM_NEGATIVE_BACTERIUM(6)	0.003
61	MYELOID_LEUKOCYTE_ACTIVATION(4)	0.003
62	IMMUNE_RESPONSE_REGULATING_SIGNALING_PATHWAY(5)	0.004
63	CYTOKINE_MEDIATED_SIGNALING_PATHWAY(6)	0.004
64	T_CELL_MIGRATION(5)	0.004
65	ACTIVATION_OF_IMMUNE_RESPONSE(3)	0.004
66	APPENDAGE_MORPHOGENESIS(4)&LIMB_MORPHOGENESIS(5)	0.004
67	EMBRYONIC_APPENDAGE_MORPHOGENESIS(5)&EMBRYONIC_LIMB_MORPHOGENESIS(6)	0.004
68	REGULATION_OF_TUMOR_NECROSIS_FACTOR_SUPERFAMILY_CYTOKINE_PRODUCTION(5)	0.004
69	REGULATION_OF_CELL_KILLING(3)	0.005
70	ANTIGEN_PROCESSING_AND_PRESENTATION_OF_EXOGENOUS_ANTIGEN(4)	0.005
71	MEMBRANE_INVAGINATION(5)	0.005
72	REGULATION_OF_LEUKOCYTE_MEDIATED_CYTOTOXICITY(4)	0.005
73	APPENDAGE_DEVELOPMENT(4)&LIMB_DEVELOPMENT(5)	0.005
74	REGULATION_OF_CYTOKINE_SECRETION(5)	0.005
75	IMMUNE_RESPONSE_ACTIVATING_SIGNAL_TRANSDUCTION(4)	0.005
76	LEUKOCYTE_MEDIATED_CYTOTOXICITY(3)	0.005
77	RESPONSE_TO_PROTOZOAN(4)	0.005
78	REGULATION_OF_TYPE_I_INTERFERON_PRODUCTION(5)	0.005
79	CELL_KILLING(2)	0.005
80	REGULATION_OF_DEFENSE_RESPONSE_TO_VIRUS(4)	0.005
81	RESPONSE_TO_INTERFERON_GAMMA(5)	0.005
82	REGULATION_OF_IMMUNE_RESPONSE(4)	0.006
83	ANTIGEN_PROCESSING_AND_PRESENTATION_OF_EXOGENOUS_PEPTIDE_ANTIGEN(5)	0.006
84	TUMOR_NECROSIS_FACTOR_SUPERFAMILY_CYTOKINE_PRODUCTION(5)	0.005
85	MALE_GONAD_DEVELOPMENT(5)&DEVELOPMENT_OF_PRIMARY_MALE_SEXUAL_CHARACTERISTICS(5)	0.006
86	TUMOR_NECROSIS_FACTOR_PRODUCTION(6)	0.006
87	MYELOID_LEUKOCYTE_MEDIATED_IMMUNITY(5)	0.007
88	REGULATION_OF_LEUKOCYTE_MEDIATED_IMMUNITY(5)	0.007
89	REGULATION_OF_ENDOCYTOSIS(4)	0.008
90	REGULATION_OF_TUMOR_NECROSIS_FACTOR_PRODUCTION(6)	0.008
91	INNATE_IMMUNE_RESPONSE_ACTIVATING_SIGNAL_TRANSDUCTION(5)	0.008
92	LYMPHOCYTE_MIGRATION(4)	0.008
93	B_CELL_MEDIATED_IMMUNITY(6)	0.008
94	POSITIVE_REGULATION_OF_INNATE_IMMUNE_RESPONSE(5)	0.008
95	LYMPHOCYTE_MEDIATED_IMMUNITY(5)	0.008
96	POSITIVE_REGULATION_OF_IMMUNE_RESPONSE(4)	0.008
97	REGULATION_OF_MYELOID_LEUKOCYTE_MEDIATED_IMMUNITY(6)	0.008
98	REGULATION_OF_IMMUNE_EFFECTOR_PROCESS(4)	0.008
99	POSITIVE_REGULATION_OF_LYMPHOCYTE_ACTIVATION(5)	0.009

100	ADAPTIVE_IMMUNE_RESPONSE_BASED_ON_SOMATIC_RECOMBINATION_OF_IMMUNE_RECEPTORS_BUILT_FROM_IMMUNOGLOBULIN_SUPERFAMILY_DOMAINS(5)	0.009
101	POSITIVE_REGULATION_OF_TUMOR_NECROSIS_FACTOR_SUPERFAMILY_CYTOKINE_PRODUCTION(5)	0.009
102	LEUKOCYTE_ACTIVATION(3)	0.010
103	FORELIMB_MORPHOGENESIS(6)	0.010
104	IMMUNOGLOBULIN_MEDIATED_IMMUNE_RESPONSE(7)	0.010
105	PATTERN_RECOGNITION_RECEPTOR_SIGNALING_PATHWAY(6)	0.010
106	NITRIC_OXIDE_METABOLIC_PROCESS(4)&REACTIVE_NITROGEN_SPECIES_METABOLIC_PROCESS(4)&NITRIC_OXIDE_BIOSYNTHETIC_PROCESS(5)	0.010
107	POSITIVE_REGULATION_OF_ENDOCYTOSIS(4)	0.010
108	POSITIVE_REGULATION_OF_REACTIVE_OXYGEN_SPECIES_METABOLIC_PROCESS(5)	0.011
109	ADAPTIVE_IMMUNE_RESPONSE(4)	0.011
110	POSITIVE_REGULATION_OF_LEUKOCYTE_ACTIVATION(4)	0.011
111	PROSTATE_GLAND_DEVELOPMENT(4)	0.011
112	REACTIVE_OXYGEN_SPECIES_BIOSYNTHETIC_PROCESS(4)	0.012
113	MULTI_ORGANISM_CELLULAR_PROCESS(3)	0.012
114	POSITIVE_REGULATION_OF_INFLAMMATORY_RESPONSE(5)	0.012
115	CELLULAR_EXTRAVASATION(4)	0.014
116	INTERFERON_BETA_PRODUCTION(6)	0.014
117	POSITIVE_REGULATION_OF_TYPE_I_INTERFERON_PRODUCTION(5)	0.014
118	POSITIVE_REGULATION_OF_INTERLEUKIN_1_PRODUCTION(5)	0.015
119	POSITIVE_REGULATION_OF_IMMUNE_EFFECTOR_PROCESS(4)	0.015
120	CENTRAL_NERVOUS_SYSTEM_PROJECTION_NEURON_AXONOGENESIS(8)	0.016
121	REGULATION_OF_OSTEOCLAST_DIFFERENTIATION(7)	0.016
122	POSITIVE_REGULATION_OF_TUMOR_NECROSIS_FACTOR_PRODUCTION(6)	0.018
123	NEGATIVE_REGULATION_OF_IMMUNE_SYSTEM_PROCESS(3)	0.018
124	REGULATION_OF_INTERFERON_BETA_PRODUCTION(6)	0.018
125	POSITIVE_REGULATION_OF_T_CELL_ACTIVATION(6)&POSITIVE_REGULATION_OF_LEUKOCYTE_CELL_CELL_ADHESION(6)	0.018
126	SUPEROXIDE_METABOLIC_PROCESS(5)	0.019
127	REGULATION_OF_MYELOID_CELL_DIFFERENTIATION(5)	0.020
128	POSITIVE_REGULATION_OF_CHEMOTAXIS(4)	0.020
129	REGULATION_OF_CELL_FATE_COMMITMENT(5)	0.021
130	POSITIVE_REGULATION_OF_PROTEIN_KINASE_B_SIGNALING(6)	0.021
131	REGULATION_OF_HEMOPOIESIS(4)	0.021
132	PROSTATE_GLAND_MORPHOGENESIS(4)	0.021
133	REGULATION_OF_LYMPHOCYTE_MEDIATED_IMMUNITY(6)	0.021
134	POSITIVE_REGULATION_OF_HOMOTYPIC_CELL_CELL_ADHESION(6)	0.022
135	REGULATION_OF_LYMPHOCYTE_ACTIVATION(5)	0.023
136	POSITIVE_REGULATION_OF_HEMOPOIESIS(4)	0.024
137	PROSTATE_GLAND_EPITHELIUM_MORPHOGENESIS(4)	0.025
138	VIRAL_PROCESS(4)	0.025
139	REGULATION_OF_SYNCYTIUM_FORMATION_BY_PLASMA_MEMBRANE_FUSION(4)	0.025
140	EMBRYONIC_DIGIT_MORPHOGENESIS(5)	0.025
141	REGULATION_OF_LEUKOCYTE_ACTIVATION(4)	0.025
142	REGULATION_OF_INFLAMMATORY_RESPONSE_TO_ANTIGENIC_STIMULUS(5)	0.025
143	OSTEOCLAST_DIFFERENTIATION(8)	0.026
144	BRANCHING_INVOLVED_IN_PROSTATE_GLAND_MORPHOGENESIS(5)	0.027
145	LYMPHOCYTE_ACTIVATION(4)	0.028
146	POSITIVE_REGULATION_OF_ADAPTIVE_IMMUNE_RESPONSE(5)&POSITIVE_REGULATION_OF_ADAPTIVE_IMMUNE_RESPONSE_BASED_ON_SOMATIC_RECOMBINATION_OF_IMMUNE_RECEPTORS_BUILT_FROM_IMMUNOGLOBULIN_SUPERFAMILY_DOMAINS(6)	0.028
147	REGULATION_OF_LEUKOCYTE_MIGRATION(4)	0.028

148	RESPONSE_TO_LIPID(5)	0.028
149	APOPTOTIC_CELL_CLEARANCE(5)	0.030
150	NEUTROPHIL_MEDIATED_IMMUNITY(6)	0.030
151	CENTRAL_NERVOUS_SYSTEM_NEURON_AXONOGENESIS(7)	0.030
152	POSITIVE_REGULATION_OF_LYMPHOCYTE_DIFFERENTIATION(6)	0.033
153	REGULATION_OF_GRANULOCYTE_CHEMOTAXIS(6)	0.033
154	AGING(4)	0.033
155	IRON_ION_TRANSPORT(8)	0.034
156	TYROSINE_PHOSPHORYLATION_OF_STAT_PROTEIN(7)	0.034
157	INFLAMMATORY_RESPONSE(5)	0.033
158	PROTEIN_KINASE_B_SIGNALING(6)	0.035
159	LEUKOCYTE_AGGREGATION(6)&LYMPHOCYTE_AGGREGATION(7)	0.035
160	SKELETAL_SYSTEM_DEVELOPMENT(5)	0.035
161	POSITIVE_REGULATION_OF_LEUKOCYTE_DIFFERENTIATION(5)	0.035
162	RECOMBINATIONAL_REPAIR(5)&DOUBLE_STRAND_BREAK_REPAIR_VIA_HOMOLOGOUS_RECOMBINATION(6)	0.036
163	T_CELL_ACTIVATION(5)&T_CELL_AGGREGATION(8)	0.036
164	CELL_ACTIVATION(4)	0.036
165	SYNCYTIUM_FORMATION_BY_PLASMA_MEMBRANE_FUSION(5)	0.036
166	CELLULAR_HORMONE_METABOLIC_PROCESS(4)	0.037
167	POSITIVE_REGULATION_OF_PROTEIN_SECRETION(5)	0.037
168	REGULATION_OF_PROTEIN_KINASE_B_SIGNALING(6)	0.037
169	REGULATION_OF_INFLAMMATORY_RESPONSE(5)	0.037
170	REGULATION_OF_LEUKOCYTE_DIFFERENTIATION(5)	0.037
171	ANTIGEN_PROCESSING_AND_PRESENTATION_OF_PEPTIDE_OR_POLYSACCHARIDE_ANTIGEN_VIA_MHC_CLASS_II(4)&ANTIGEN_PROCESSING_AND_PRESENTATION_OF_PEPTIDE_ANTIGEN_VIA_MHC_CLASS_II(5)	0.037
172	POSITIVE_REGULATION_OF_CELL_ACTIVATION(4)	0.037
173	EMBRYONIC_FORELIMB_MORPHOGENESIS(7)	0.037
174	POSITIVE_REGULATION_OF_LEUKOCYTE_MEDIATED_IMMUNITY(5)	0.037
175	GENITALIA_DEVELOPMENT(4)	0.038
176	INTERLEUKIN_1_PRODUCTION(5)	0.040
177	CELLULAR_RESPONSE_TO_ESTROGEN_STIMULUS(7)	0.041
178	TRABECULA_MORPHOGENESIS(4)	0.042
179	B_CELL_DIFFERENTIATION(6)	0.042
180	POSITIVE_REGULATION_OF_INTERFERON_GAMMA_PRODUCTION(5)	0.044
181	POSITIVE_REGULATION_OF_LEUKOCYTE_MEDIATED_CYTOTOXICITY(4)	0.046
182	REGULATION_OF_ADAPTIVE_IMMUNE_RESPONSE_BASED_ON_SOMATIC_RECOMBINATION_OF_IMMUNE_RECEPTORS_BUILT_FROM_IMMUNOGLOBULIN_SUPERFAMILY_DOMAINS(6)	0.046
183	REGULATION_OF_ADAPTIVE_IMMUNE_RESPONSE(5)	0.047
184	NEGATIVE_REGULATION_OF_LEUKOCYTE_ACTIVATION(4)	0.047
185	CELL_ACTIVATION_INVOLVED_IN_IMMUNE_RESPONSE(4)&LEUKOCYTE_ACTIVATION_INVOLVED_IN_IMMUNE_RESPONSE(4)	0.047
186	NEGATIVE_REGULATION_OF_DEFENSE_RESPONSE(4)	0.048
187	POSITIVE_REGULATION_OF_CELL_CELL_ADHESION(5)	0.047
188	REGULATION_OF_RESPONSE_TO_EXTERNAL_STIMULUS(4)	0.048
189	REGULATION_OF_MYELOID_LEUKOCYTE_DIFFERENTIATION(6)	0.049
190	INTERLEUKIN_1_BETA_PRODUCTION(6)	0.049
191	MALE_GENITALIA_DEVELOPMENT(5)	0.049
192	NEGATIVE_REGULATION_OF_LEUKOCYTE_APOPTOTIC_PROCESS(7)	0.049
