# Supplementary material for: E2F1 acetylation directs p300/CBP-mediated histone acetylation at DNA double-strand breaks to facilitate repair
Source: Nat Commun. 2019 Oct 30;10:4951. doi: 10.1038/s41467-019-12861-8 (PMC6821830; doi:10.1038/s41467-019-12861-8)
Supplement: Supplementary file 8 — Supplementary Data 4 [file 41467_2019_12861_MOESM8_ESM.pdf]

Supplementary Data 4: List of gene sets significantly enriched in *E2f1*$^{3KR/3KR}$ compared to wild type MEFs after DNA damage. False Discovery Rate (FDR), q value ≤ 0.05.

#	NAME	FDR q-value
1	CELL_CHEMOTAXIS(5)	0.000
2	RESPONSE_TO_OTHER_ORGANISM(3)&RESPONSE_TO_EXTERNAL_BIOTIC_STIMULUS(4)	0.000
3	LEUKOCYTE_CHEMOTAXIS(4)	0.000
4	RESPONSE_TO_BIOTIC_STIMULUS(3)	0.000
5	DEFENSE_RESPONSE_TO_OTHER_ORGANISM(4)	0.000
6	RESPONSE_TO_VIRUS(4)	0.000
7	MYELOID_LEUKOCYTE_MIGRATION(4)	0.000
8	GRANULOCYTE_MIGRATION(5)	0.000
9	POSITIVE_REGULATION_OF_PHAGOCYTOSIS(5)	0.000
10	DEFENSE_RESPONSE_TO_BACTERIUM(5)	0.000
11	RESPONSE_TO_BACTERIUM(4)	0.000
12	REGULATION_OF_PHAGOCYTOSIS(5)	0.000
13	NEUTROPHIL_MIGRATION(6)	0.000
14	REGULATION_OF_RESPONSE_TO_BIOTIC_STIMULUS(4)	0.000
15	GRANULOCYTE_CHEMOTAXIS(5)	0.000
16	NEUTROPHIL_CHEMOTAXIS(6)	0.000
17	POSITIVE_REGULATION_OF_CYTOKINE_PRODUCTION(4)	0.000
18	LEUKOCYTE_MIGRATION(3)	0.001
19	REGULATION_OF_MULTI_ORGANISM_PROCESS(3)	0.001
20	CYTOKINE_PRODUCTION(4)	0.001
21	EMBRYONIC_APPENDAGE_MORPHOGENESIS(5)&EMBRYONIC_LIMB_MORPHOGENESIS(6)	0.001
22	REGULATION_OF_B_CELL_DIFFERENTIATION(7)	0.002
23	REGULATION_OF_LEUKOCYTE_MEDIATED_CYTOTOXICITY(4)	0.002
24	FORELIMB_MORPHOGENESIS(6)	0.002
25	REGULATION_OF_CYTOKINE_PRODUCTION(4)	0.002
26	REGULATION_OF_CELL_KILLING(3)	0.002
27	APPENDAGE_MORPHOGENESIS(4)&LIMB_MORPHOGENESIS(5)	0.002
28	REGULATION_OF_OSTEOCLAST_DIFFERENTIATION(7)	0.003
29	HEART_TRABECULA_MORPHOGENESIS(5)	0.003
30	IMMUNE_EFFECTOR_PROCESS(3)	0.003
31	APPENDAGE_DEVELOPMENT(4)&LIMB_DEVELOPMENT(5)	0.003
32	POSITIVE_REGULATION_OF_RESPONSE_TO_EXTERNAL_STIMULUS(4)	0.003
33	DEFENSE_RESPONSE_TO_PROTOZOAN(5)	0.004
34	POSITIVE_REGULATION_OF_DEFENSE_RESPONSE(4)	0.004
35	RESPONSE_TO_CYTOKINE(5)	0.004
36	NEGATIVE_REGULATION_OF_MULTI_ORGANISM_PROCESS(3)	0.004
37	REGULATION_OF_LEUKOCYTE_MEDIATED_IMMUNITY(5)	0.004
38	LEUKOCYTE_MEDIATED_IMMUNITY(4)	0.004
39	PHAGOCYTOSIS(4)	0.004
40	CELLULAR_RESPONSE_TO_CYTOKINE_STIMULUS(6)	0.004
41	EMBRYONIC_DIGIT_MORPHOGENESIS(5)	0.004
42	REGULATION_OF_SYMBIOSIS_ENCOMPASSING_MUTUALISM_THROUGH_PARASITISM(4)	0.004
43	POSITIVE_REGULATION_OF_IMMUNE_SYSTEM_PROCESS(3)	0.004
44	INTERSPECIES_INTERACTION_BETWEEN_ORGANISMS(3)&SYMBIOSIS_ENCOMPASSING_MUTUALISM_THROUGH_PARASITISM(4)	0.005
45	MALE_SEX_DIFFERENTIATION(5)	0.005
46	MALE_GONAD_DEVELOPMENT(5)&DEVELOPMENT_OF_PRIMARY_MALE_SEXUAL_CHARACTE	0.005

	RISTICS(5)	
47	POSITIVE_REGULATION_OF_INTERFERON_GAMMA_PRODUCTION(5)	0.005
48	RESPONSE_TO_INTERFERON_BETA(6)	0.005
49	DEFENSE_RESPONSE_TO_GRAM_POSITIVE_BACTERIUM(6)	0.005
50	POSITIVE_REGULATION_OF_LEUKOCYTE_MIGRATION(4)	0.005
51	T_CELL_MIGRATION(5)	0.005
52	DEFENSE_RESPONSE(4)	0.005
53	RESPONSE_TO_MOLECULE_OF_BACTERIAL_ORIGIN(5)	0.005
54	OSTEOCLAST_DIFFERENTIATION(8)	0.005
55	LYMPHOCYTE_MEDIATED_IMMUNITY(5)	0.005
56	MYELOID_LEUKOCYTE_MEDIATED_IMMUNITY(5)	0.005
57	POSITIVE_REGULATION_OF_HEMOPOIESIS(4)	0.006
58	IMMUNE_RESPONSE(3)	0.006
59	POSITIVE_REGULATION_OF_LEUKOCYTE_MEDIATED_IMMUNITY(5)	0.006
60	POSITIVE_REGULATION_OF_LEUKOCYTE_DIFFERENTIATION(5)	0.006
61	LYMPHOCYTE_MIGRATION(4)	0.007
62	REGULATION_OF_LYMPHOCYTE_MEDIATED_IMMUNITY(6)	0.007
63	POSITIVE_REGULATION_OF_LEUKOCYTE_CHEMOTAXIS(5)	0.008
64	POSITIVE_REGULATION_OF_IMMUNE_EFFECTOR_PROCESS(4)	0.008
65	REGULATION_OF_DEFENSE_RESPONSE_TO_VIRUS(4)	0.008
66	CELLULAR_RESPONSE_TO_INTERFERON_BETA(7)	0.008
67	INNATE_IMMUNE_RESPONSE(4)	0.008
68	REGULATION_OF_IMMUNE_SYSTEM_PROCESS(3)	0.009
69	CYTOKINE_SECRETION(5)	0.010
70	POSITIVE_REGULATION_OF_CYTOKINE_SECRETION(5)	0.010
71	LEUKOCYTE_MEDIATED_CYTOTOXICITY(3)	0.010
72	POSITIVE_REGULATION_OF_PROTEIN_SECRETION(5)	0.010
73	B_CELL_MEDIATED_IMMUNITY(6)	0.011
74	POSITIVE_REGULATION_OF_MYELOID_CELL_DIFFERENTIATION(5)	0.012
75	CELLULAR_EXTRAVASATION(4)	0.013
76	REGULATION_OF_DEFENSE_RESPONSE(5)	0.013
77	REGULATION_OF_LEUKOCYTE_CHEMOTAXIS(5)	0.013
78	REGULATION_OF_LEUKOCYTE_DIFFERENTIATION(5)	0.013
79	REGULATION_OF_MYELOID_LEUKOCYTE_DIFFERENTIATION(6)	0.013
80	ADAPTIVE_IMMUNE_RESPONSE_BASED_ON_SOMATIC_RECOMBINATION_OF_IMMUNE_RECEPTORS_BUILT_FROM_IMMUNOGLOBULIN_SUPERFAMILY_DOMAINS(5)	0.013
81	POSITIVE_REGULATION_OF_MYELOID_LEUKOCYTE_DIFFERENTIATION(6)	0.013
82	SYNCYTIUM_FORMATION_BY_PLASMA_MEMBRANE_FUSION(5)	0.013
83	RESPONSE_TO_PROTOZOAN(4)	0.013
84	MYELOID_DENDRITIC_CELL_ACTIVATION(5)	0.015
85	REGULATION_OF_MYELOID_CELL_DIFFERENTIATION(5)	0.015
86	POSITIVE_REGULATION_OF_ADAPTIVE_IMMUNE_RESPONSE(5)&POSITIVE_REGULATION_OF_ADAPTIVE_IMMUNE_RESPONSE_BASED_ON_SOMATIC_RECOMBINATION_OF_IMMUNE_RECEPTORS_BUILT_FROM_IMMUNOGLOBULIN_SUPERFAMILY_DOMAINS(6)	0.015
87	EMBRYONIC_FORELIMB_MORPHOGENESIS(7)	0.015
88	CELL_KILLING(2)	0.015
89	ADAPTIVE_IMMUNE_RESPONSE(4)	0.015
90	REGULATION_OF_MYELOID_LEUKOCYTE_MEDIATED_IMMUNITY(6)	0.016
91	B_CELL_DIFFERENTIATION(6)	0.016
92	REGULATION_OF_TUMOR_NECROSIS_FACTOR_SUPERFAMILY_CYTOKINE_PRODUCTION(5)	0.016
93	REGULATION_OF_HEMOPOIESIS(4)	0.016
94	CYTOKINE_MEDIATED_SIGNALING_PATHWAY(6)	0.016
95	LEUKOCYTE_ACTIVATION(3)	0.016
96	POSITIVE_REGULATION_OF_LYMPHOCYTE_MEDIATED_IMMUNITY(6)	0.017

97	TUMOR_NECROSIS_FACTOR_SUPERFAMILY_CYTOKINE_PRODUCTION(5)	0.017
98	SUPEROXIDE_METABOLIC_PROCESS(5)	0.017
99	REGULATION_OF_INNATE_IMMUNE_RESPONSE(5)	0.016
100	REGULATION_OF_IMMUNE_RESPONSE(4)	0.016
101	POSITIVE_REGULATION_OF_REACTIVE_OXYGEN_SPECIES_METABOLIC_PROCESS(5)	0.017
102	REGULATION_OF_INTERLEUKIN_12_PRODUCTION(5)	0.018
103	REGULATION_OF_IMMUNE_EFFECTOR_PROCESS(4)	0.018
104	SYNCYTIUM_FORMATION(4)	0.019
105	ACTIVATION_OF_INNATE_IMMUNE_RESPONSE(4)	0.019
106	POSITIVE_REGULATION_OF_LEUKOCYTE_ACTIVATION(4)	0.019
107	IMMUNOGLOBULIN_MEDIATED_IMMUNE_RESPONSE(7)	0.020
108	NEUTROPHIL_MEDIATED_IMMUNITY(6)	0.021
109	DEFENSE_RESPONSE_TO_VIRUS(4)	0.021
110	DEVELOPMENT_OF_PRIMARY_SEXUAL_CHARACTERISTICS(4)	0.021
111	RESPONSE_TO_LIPOPOLYSACCHARIDE(5)	0.021
112	TUMOR_NECROSIS_FACTOR_PRODUCTION(6)	0.022
113	TYPE_I_INTERFERON_PRODUCTION(5)	0.021
114	GONAD_DEVELOPMENT(4)	0.021
115	POSITIVE_REGULATION_OF_LYMPHOCYTE_ACTIVATION(5)	0.022
116	POSITIVE_REGULATION_OF_TUMOR_NECROSIS_FACTOR_SUPERFAMILY_CYTOKINE_PRODUCTION(5)	0.022
117	POSITIVE_REGULATION_OF_LYMPHOCYTE_DIFFERENTIATION(6)	0.022
118	CHROMOSOME_SEGREGATION(4)	0.022
119	REGULATION_OF_INFLAMMATORY_RESPONSE_TO_ANTIGENIC_STIMULUS(5)	0.023
120	IMMUNE_RESPONSE_REGULATING_SIGNALING_PATHWAY(5)	0.023
121	DEFENSE_RESPONSE_TO_GRAM_NEGATIVE_BACTERIUM(6)	0.024
122	BRANCHING_INVOLVED_IN_PROSTATE_GLAND_MORPHOGENESIS(5)	0.025
123	IMMUNE_RESPONSE_ACTIVATING_SIGNAL_TRANSDUCTION(4)	0.025
124	REGULATION_OF_TUMOR_NECROSIS_FACTOR_PRODUCTION(6)	0.026
125	INTERFERON_GAMMA_PRODUCTION(5)	0.025
126	LYMPHOCYTE_ACTIVATION(4)	0.026
127	REGULATION_OF_ADAPTIVE_IMMUNE_RESPONSE_BASED_ON_SOMATIC_RECOMBINATION_OF_IMMUNE_RECEPTORS_BUILT_FROM_IMMUNOGLOBULIN_SUPERFAMILY_DOMAINS(6)	0.026
128	LEUKOCYTE_DIFFERENTIATION(6)	0.026
129	POSITIVE_REGULATION_OF_IMMUNE_RESPONSE(4)	0.026
130	POSITIVE_REGULATION_OF_ENDOCYTOSIS(4)	0.026
131	SKELETAL_SYSTEM_DEVELOPMENT(5)	0.026
132	POSITIVE_REGULATION_OF_T_CELL_ACTIVATION(6)&POSITIVE_REGULATION_OF_LEUKOCYTE_CELL_CELL_ADHESION(6)	0.027
133	PHAGOCYTOSIS_ENGULFMENT(5)	0.027
134	INTERLEUKIN_12_PRODUCTION(5)	0.027
135	APOPTOTIC_CELL_CLEARANCE(5)	0.028
136	REGULATION_OF_T_CELL_MEDIATED_IMMUNITY(7)	0.029
137	REGULATION_OF_ADAPTIVE_IMMUNE_RESPONSE(5)	0.030
138	REGULATION_OF_LYMPHOCYTE_ACTIVATION(5)	0.030
139	POSITIVE_REGULATION_OF_CHEMOTAXIS(4)	0.032
140	T_CELL_ACTIVATION(5)&T_CELL_AGGREGATION(8)	0.032
141	ACTIVATION_OF_IMMUNE_RESPONSE(3)	0.032
142	CELL_ACTIVATION(4)	0.032
143	ANTIGEN_PROCESSING_AND_PRESENTATION(3)	0.032
144	REGULATION_OF_SYNCYTIUM_FORMATION_BY_PLASMA_MEMBRANE_FUSION(4)	0.032
145	MYELOID_LEUKOCYTE_ACTIVATION(4)	0.033
146	POSITIVE_REGULATION_OF_INFLAMMATORY_RESPONSE(5)	0.034
147	INFLAMMATORY_RESPONSE_TO_ANTIGENIC_STIMULUS(4)	0.034

148	REGULATION_OF_INTERFERON_GAMMA_PRODUCTION(5)	0.034
149	NITRIC_OXIDE_METABOLIC_PROCESS(4)&REACTIVE_NITROGEN_SPECIES_METABOLIC_PROCESS(4)&NITRIC_OXIDE_BIOSYNTHETIC_PROCESS(5)	0.035
150	REGULATION_OF_LYMPHOCYTE_DIFFERENTIATION(6)	0.036
151	LYMPHOCYTE_DIFFERENTIATION(5)	0.037
152	PROXIMAL_DISTAL_PATTERN_FORMATION(6)	0.037
153	MEIOSIS_I(5)	0.037
154	CELL_ACTIVATION_INVOLVED_IN_IMMUNE_RESPONSE(4)&LEUKOCYTE_ACTIVATION_INVOLVED_IN_IMMUNE_RESPONSE(4)	0.037
155	DNA_DEPENDENT_DNA_REPLICATION(7)	0.038
156	LEUKOCYTE_AGGREGATION(6)&LYMPHOCYTE_AGGREGATION(7)	0.038
157	POSITIVE_REGULATION_OF_INNATE_IMMUNE_RESPONSE(5)	0.038
158	REGULATION_OF_T_CELL_ACTIVATION(6)&REGULATION_OF_LEUKOCYTE_CELL_CELL_ADHESION(6)	0.038
159	POSITIVE_REGULATION_OF_TUMOR_NECROSIS_FACTOR_PRODUCTION(6)	0.039
160	TRABECULA_MORPHOGENESIS(4)	0.039
161	POSITIVE_REGULATION_OF_TYPE_I_INTERFERON_PRODUCTION(5)	0.039
162	POSITIVE_REGULATION_OF_LEUKOCYTE_MEDIATED_CYTOTOXICITY(4)	0.039
163	MEMBRANE_INVAGINATION(5)	0.040
164	CYTOKINE_METABOLIC_PROCESS(5)	0.042
165	REGULATION_OF_LEUKOCYTE_ACTIVATION(4)	0.042
166	POSITIVE_REGULATION_OF_HOMOTYPIC_CELL_CELL_ADHESION(6)	0.042
167	CARTILAGE_DEVELOPMENT(5)	0.042
168	B_CELL_ACTIVATION_INVOLVED_IN_IMMUNE_RESPONSE(6)	0.043
169	MYOBLAST_FUSION(6)	0.043
170	LEUKOCYTE_CELL_CELL_ADHESION(5)	0.043
171	POSITIVE_REGULATION_OF_CELL_ACTIVATION(4)	0.044
172	NEGATIVE_REGULATION_OF_LEUKOCYTE_ACTIVATION(4)	0.044
173	REGULATION_OF_LEUKOCYTE_MIGRATION(4)	0.044
174	NEGATIVE_REGULATION_OF_IMMUNE_SYSTEM_PROCESS(3)	0.045
175	INNATE_IMMUNE_RESPONSE_ACTIVATING_SIGNAL_TRANSDUCTION(5)	0.046
176	REGULATION_OF_TISSUE_REMODELING(4)	0.046
177	REGULATION_OF_CYTOKINE_SECRETION(5)	0.047
178	REGULATION_OF_TYPE_I_INTERFERON_PRODUCTION(5)	0.047
179	CHONDROCYTE_DIFFERENTIATION(6)	0.047
180	REGULATION_OF_REACTIVE_OXYGEN_SPECIES_BIOSYNTHETIC_PROCESS(5)	0.048
181	POSITIVE_REGULATION_OF_PROTEIN_TRANSPORT(4)	0.049
182	SEX_DIFFERENTIATION(4)	0.049
