# Supplementary material for: E2F1 acetylation directs p300/CBP-mediated histone acetylation at DNA double-strand breaks to facilitate repair
Source: Nat Commun. 2019 Oct 30;10:4951. doi: 10.1038/s41467-019-12861-8 (PMC6821830; doi:10.1038/s41467-019-12861-8)
Supplement: Supplementary file 9 — Supplementary Data 5 [file 41467_2019_12861_MOESM9_ESM.pdf]

Supplementary Data 5_Cover Page

E2F1 Acetylation Directs p300/CBP-mediated Histone Acetylation at DNA Double-Strand Breaks to Facilitate Repair.

Manickavinayaham et al.

Uncropped and unprocessed scans of blots/gels are provided as Supplementary Data 5. The size of this file is more than 30 MB. Thus, it is not included in Supplementary Information, instead provided as Supplementary Data 5. It includes the uncropped and unprocessed scans of blots/gels corresponding to

Main Figures - 1b, c; 2a, b; 4b, c; 5b; 6b, d

and

Supplementary Figures - 1c; 2a; 3d; 4b; 6e; 7a, b

Fig. 1b

Source

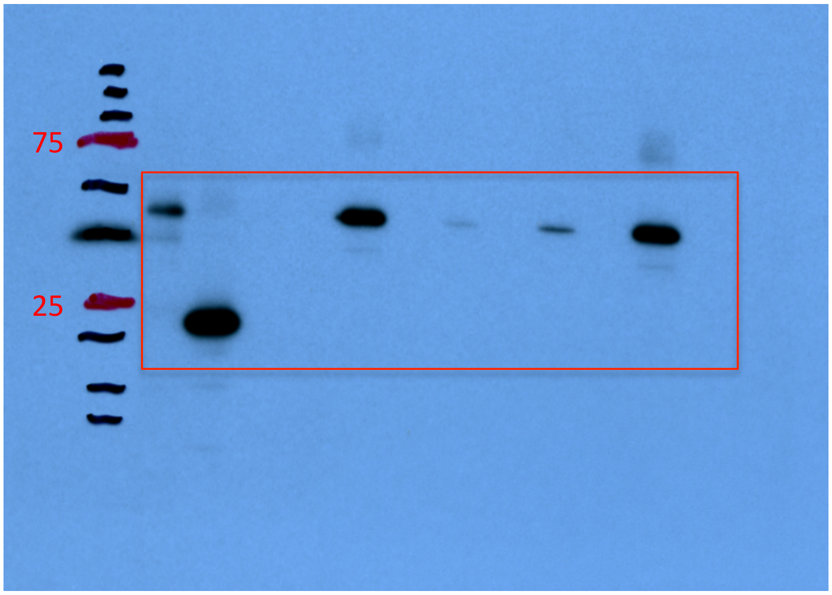

Cropped

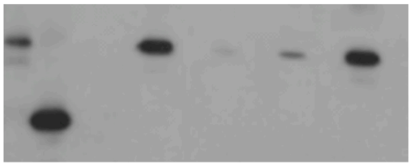

Molecular weight markers in red are 75 KD (Top) and 25 KD (Bottom)

Fig. 1c

Source

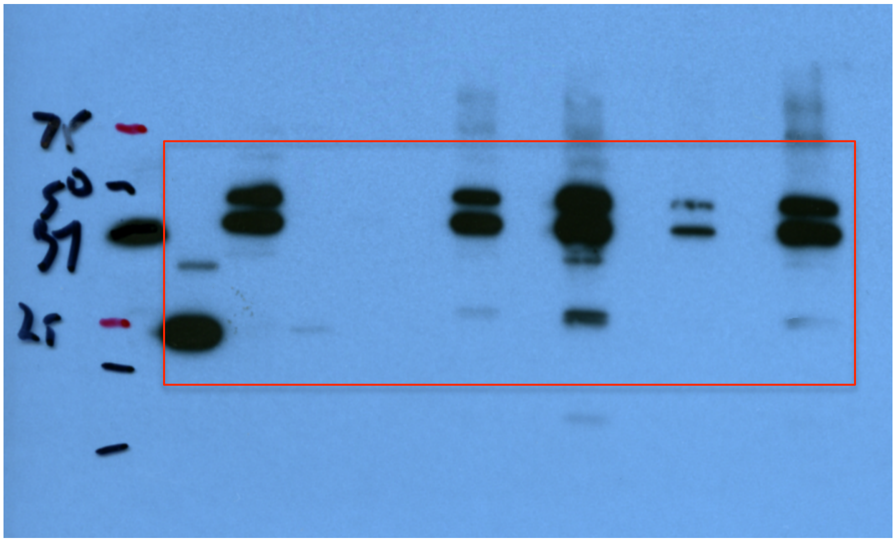

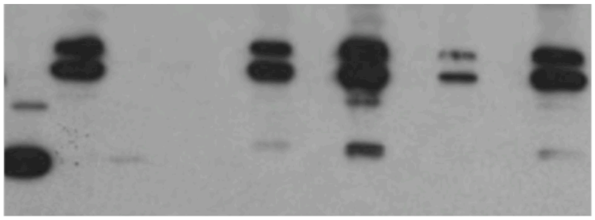

Cropped

Fig. 2a

Input

α-E2F1
(60 KD)

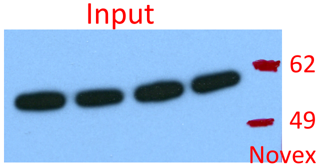

M - Molecular weight markers (KD)

α-E2F1
(60 KD)

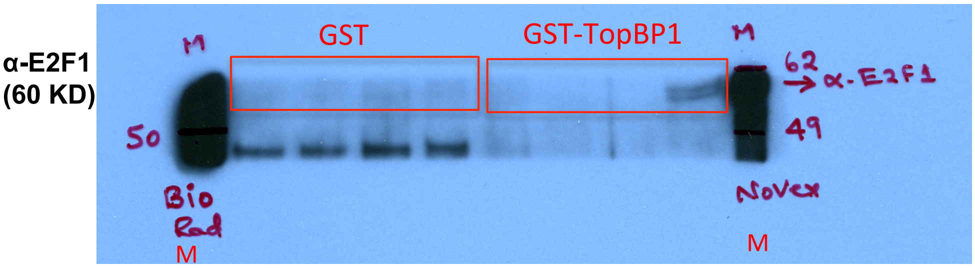

Not used in paper – After pull down with beads, sup was checked for pull down efficiency

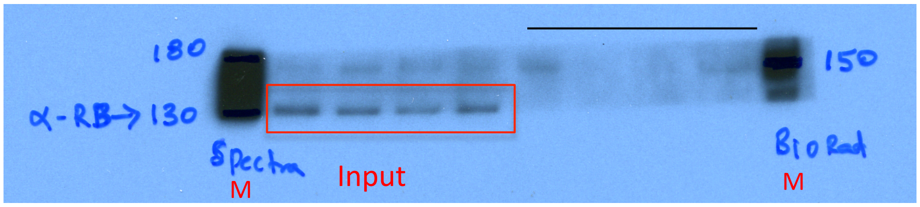

α-RB (approx. 130 KD)

M

GST

GST-TopBP1

M

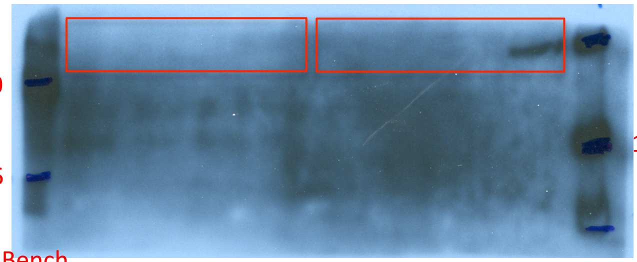

120

85

α-RB (approx. 130 KD)

Bench Mark

Spectra

Fig. 2a continued

α-p300 (300 KD)

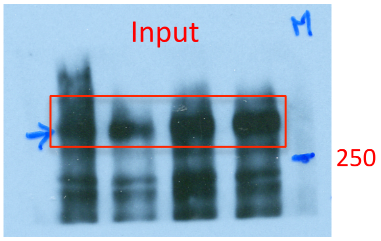

α-p300 (300 KD)

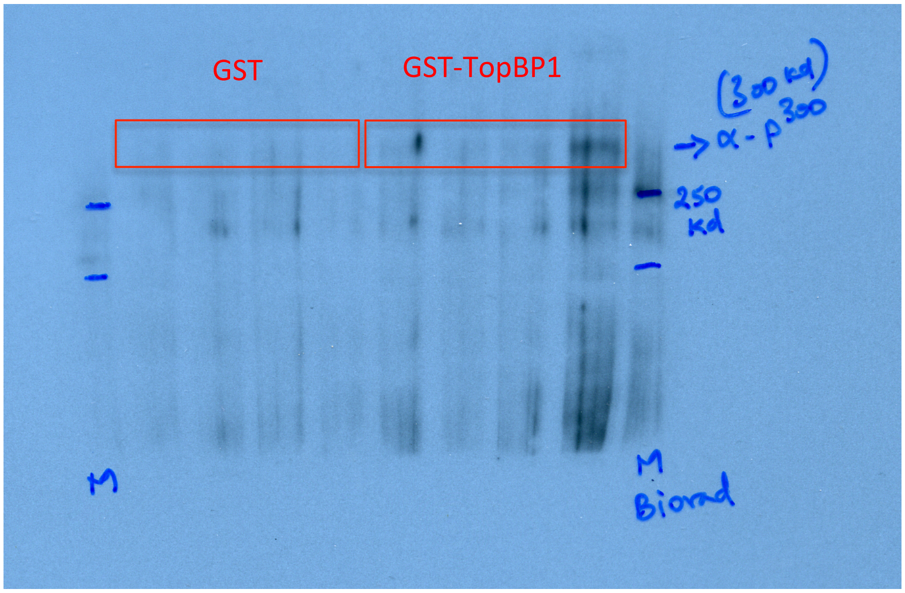

α-CBP (265 KD)

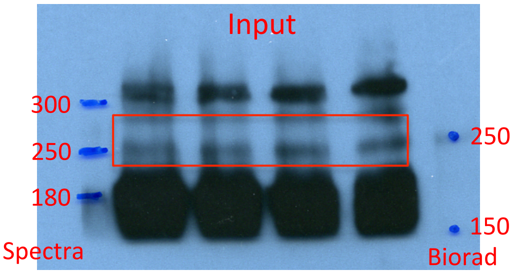

α-CBP (265 KD)

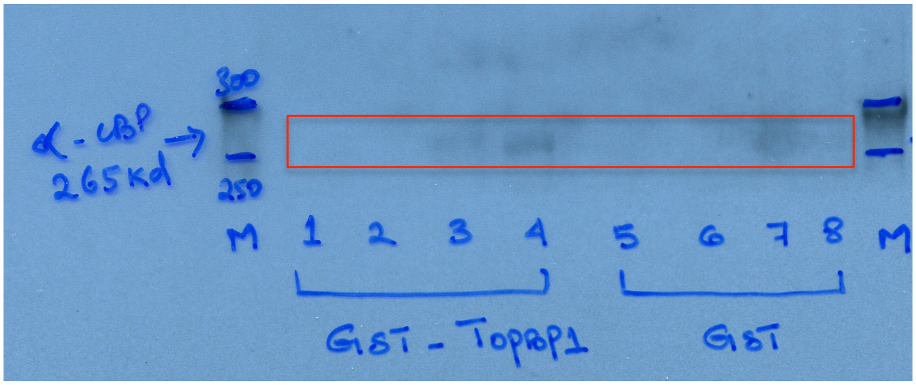

Fig. 2b

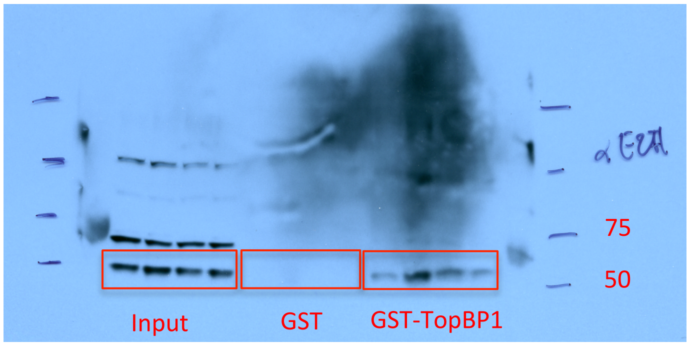

α-E2F1 (60 KD)

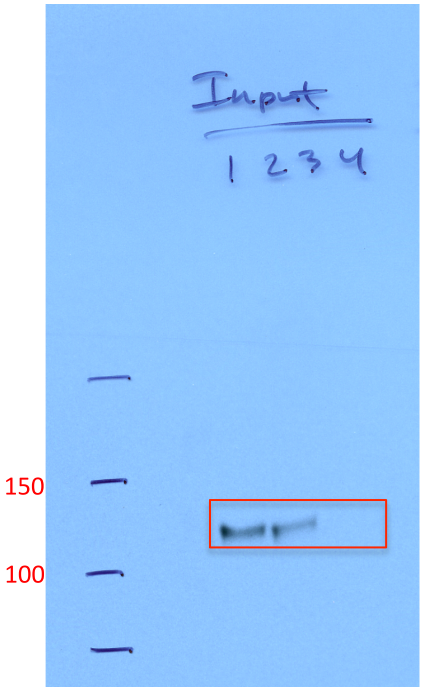

α-RB (approx.
130 KD)

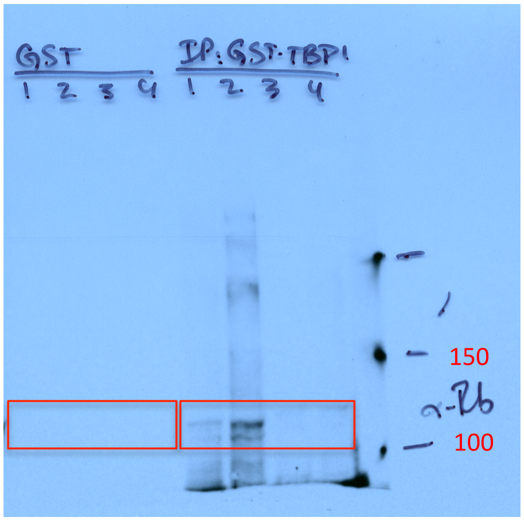

α-CBP (265 KD)

α-p300 (300 KD)

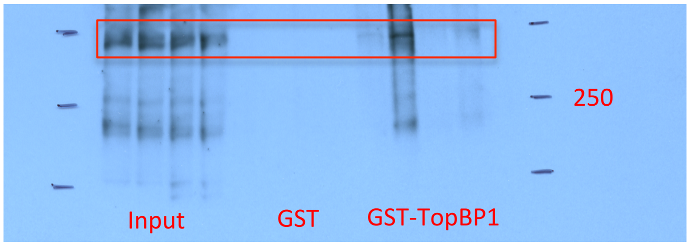

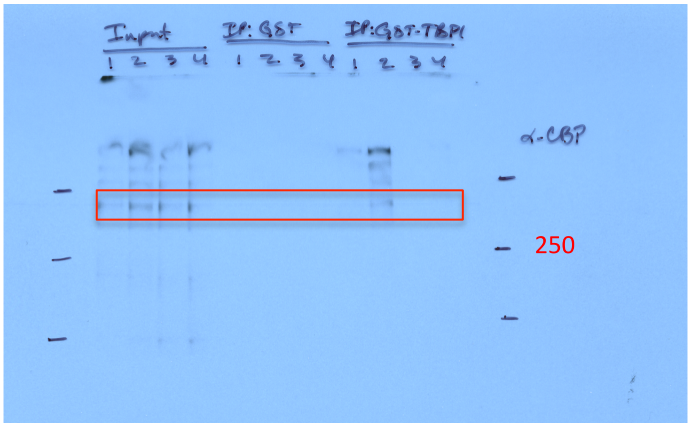

Fig. 4b

α-E2F1
(60 KD)

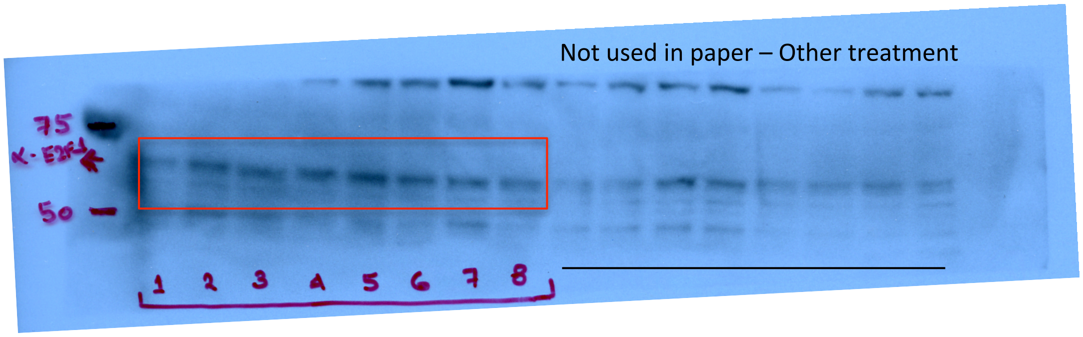

Lanes 1 – 4 WT , NCS - 0, 1, 3, 5 h
Lanes 5 – 8 3KR, NCS - 0, 1, 3, 5 h

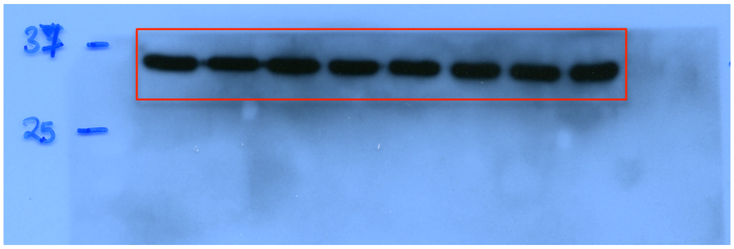

α-GAPDH
(38 KD)

Fig. 4c

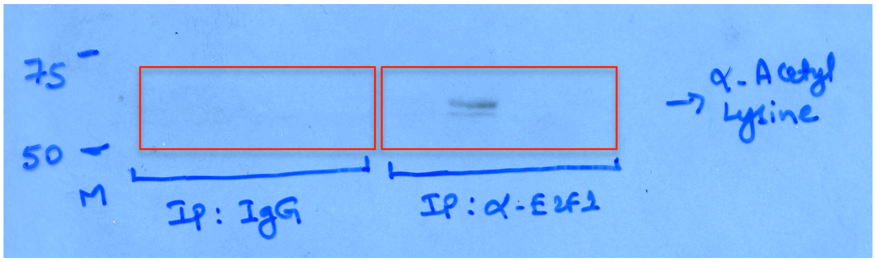

α-Ac Lysine

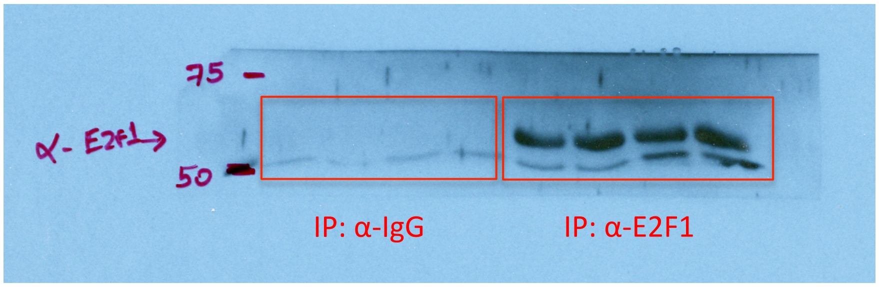

α-E2F1
(60 KD)

Fig. 5b

C – Control lane, not used in paper.
WT – IR treated lysate.

C

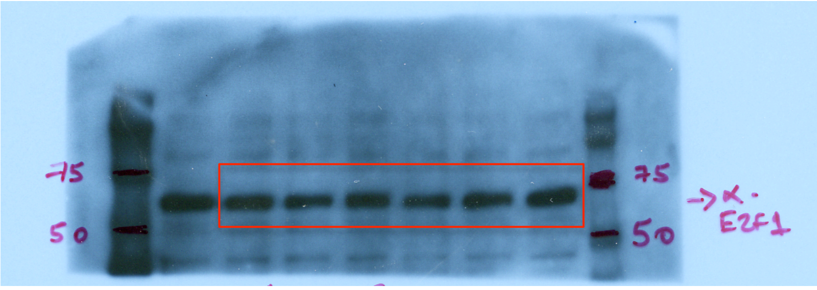

α-E2F1
(60 KD)

C

α-RB (approx. 130 KD)

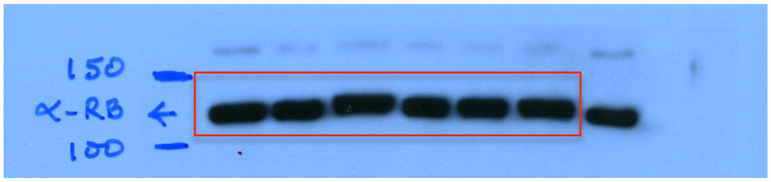

C C

α-p300
(300 KD)

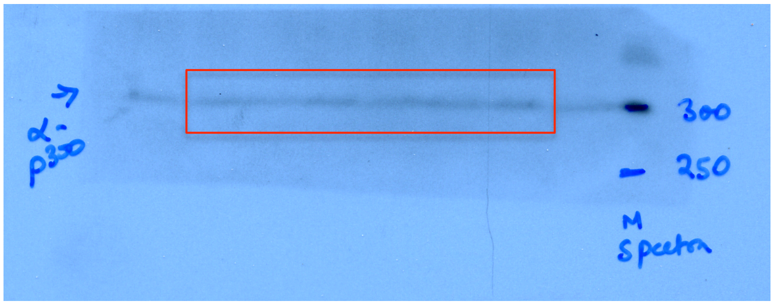

Fig. 5b continued

C – Control lane, not used in paper.
WT – IR treated lysate.

C

α-CBP
(265 KD)

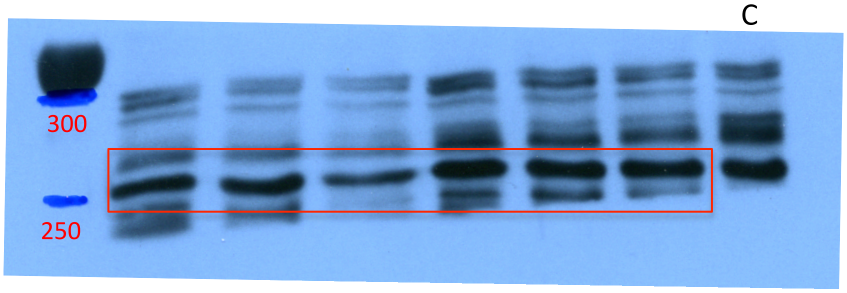

C

α-Tip60
(60 KD)

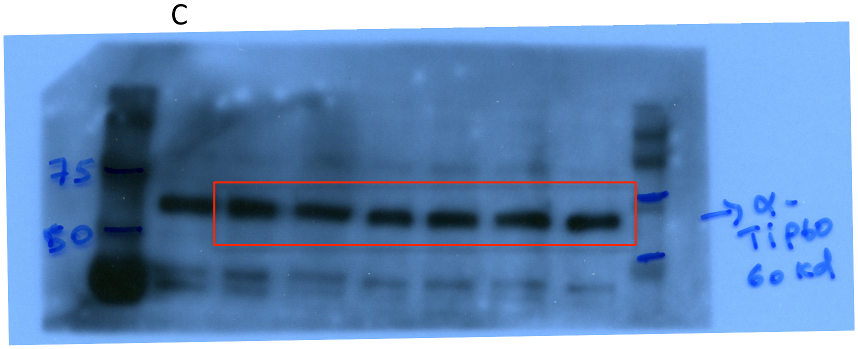

C

α-GAPDH
(38 KD)

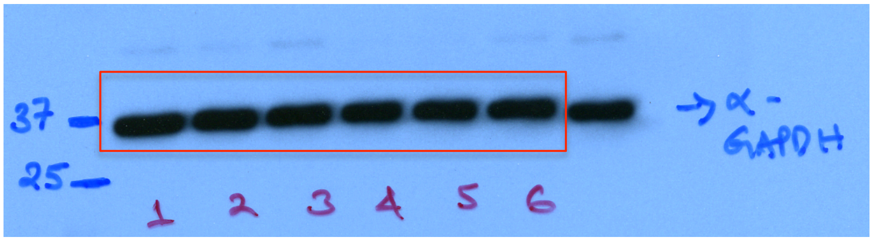

Fig. 6b

α-E2F1
(60 KD)

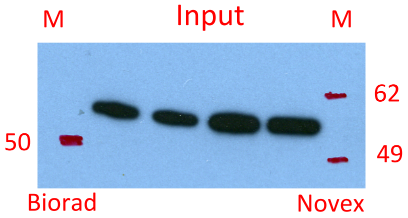

M - Molecular weight markers (KD)

α-E2F1
(60 KD)

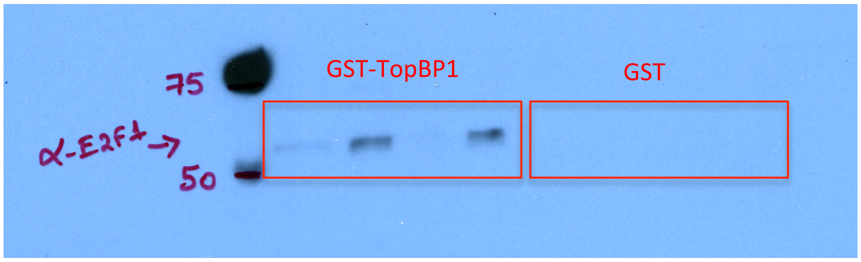

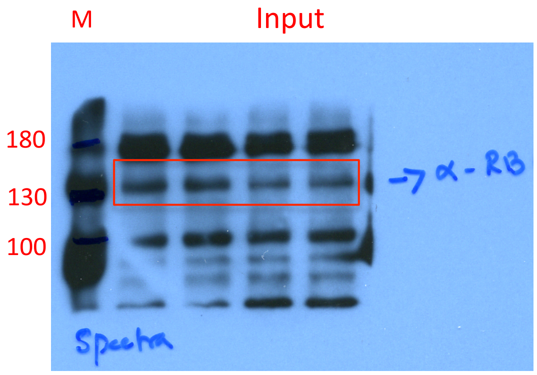

α-RB (approx. 130 KD)

α-RB (approx. 130 KD)

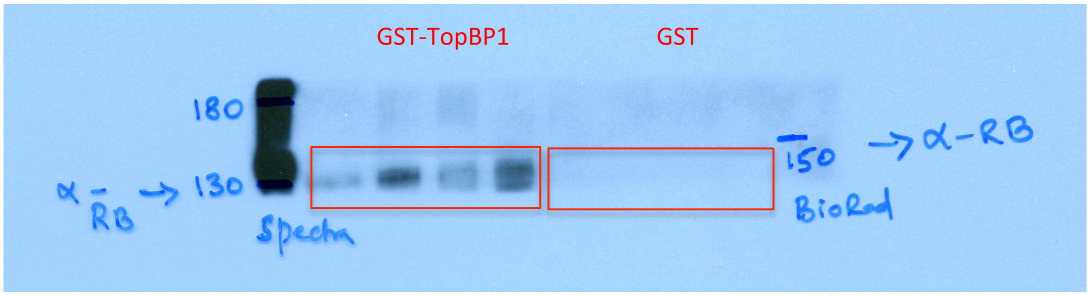

Fig. 6b continued

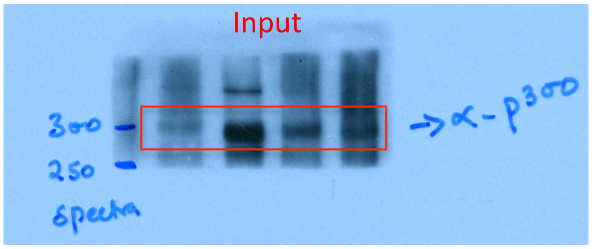

α-p300 (300 KD)

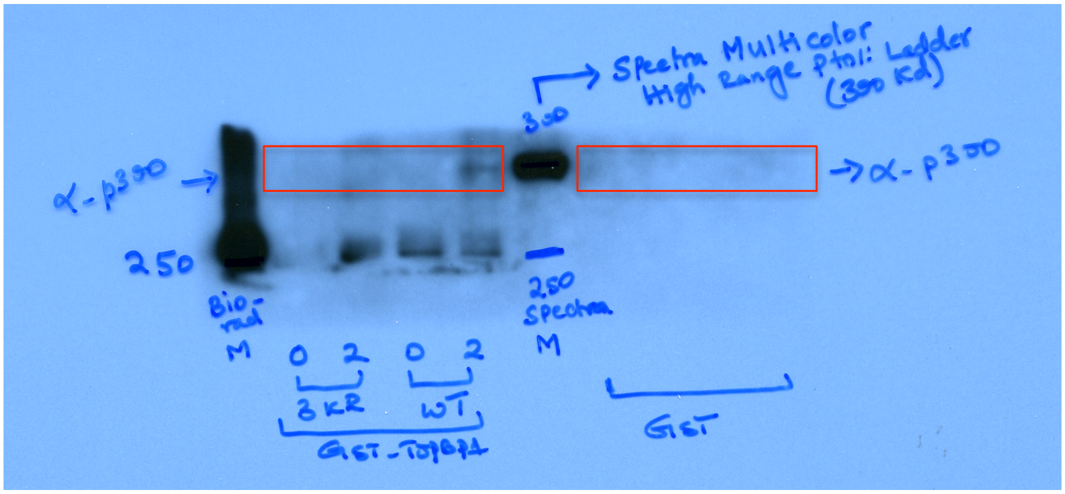

α-p300 (300 KD)

Input

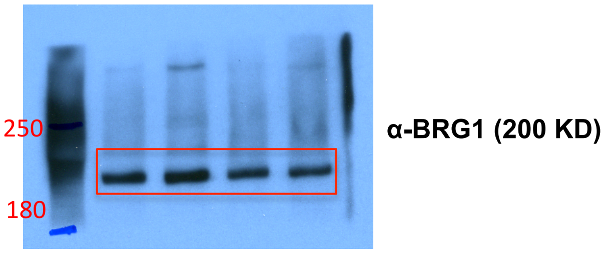

α-BRG1 (200 KD)

α-BRG1 (200 KD)

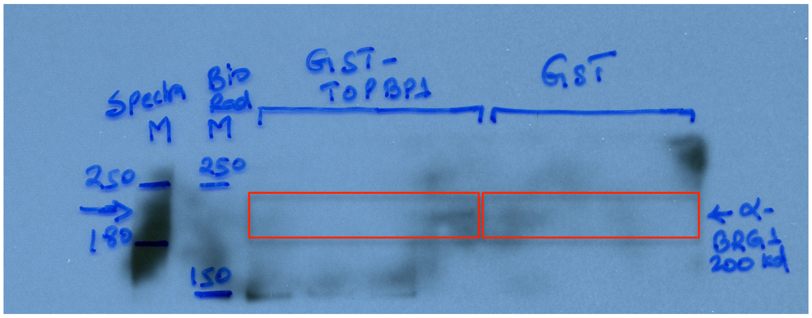

Fig. 6d

Lanes 1 – 3 WT , NCS - 0, 1, 3 h
Lanes 5 – 7 3KR, NCS - 0, 1, 3 h

Lanes 4 and 8 – WT & 3KR respectively, later time point of NCS treatment, not used in paper

α-pATM (370 KD)

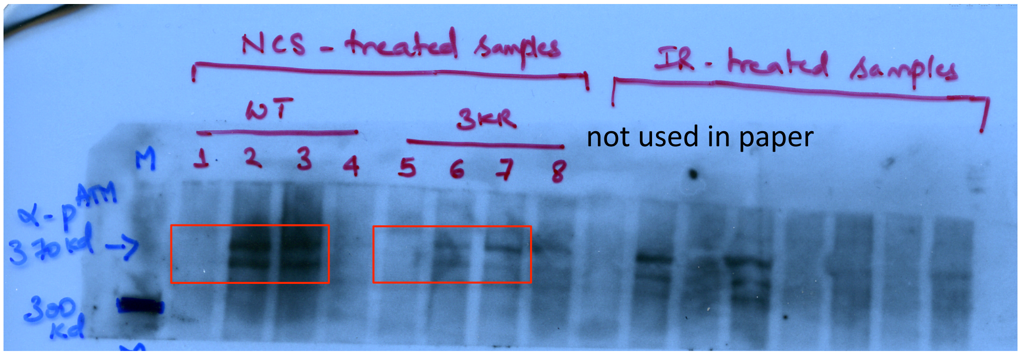

PC – Positive control, WT treated with saturated concentration of NCS, not used in paper

α-phospho p53 (53 KD)

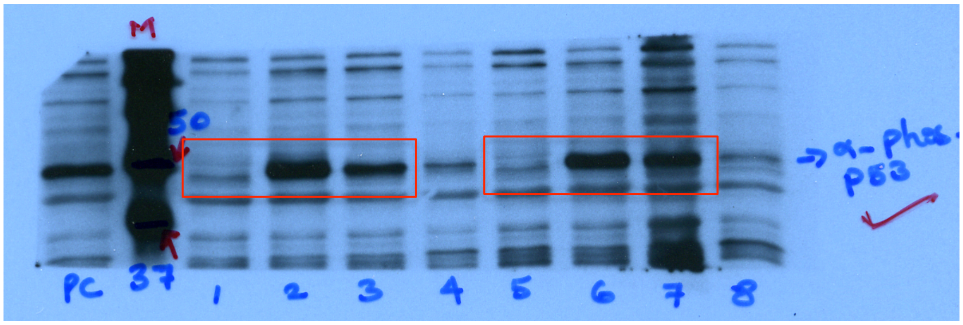

α-γH2AX (15 KD)

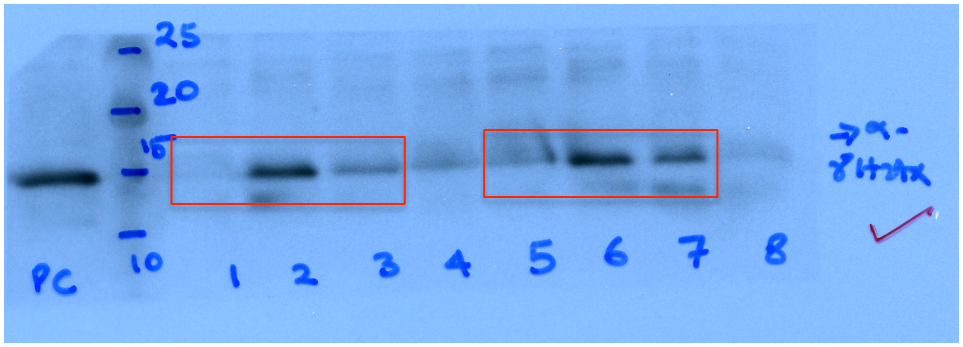

α-GAPDH (38 KD)

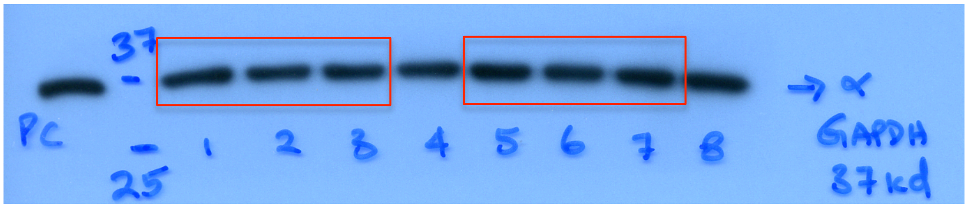

Supplementary Figure 1c

Top Panel : GST-p300 incubation with E2F1 di-acetylated peptides

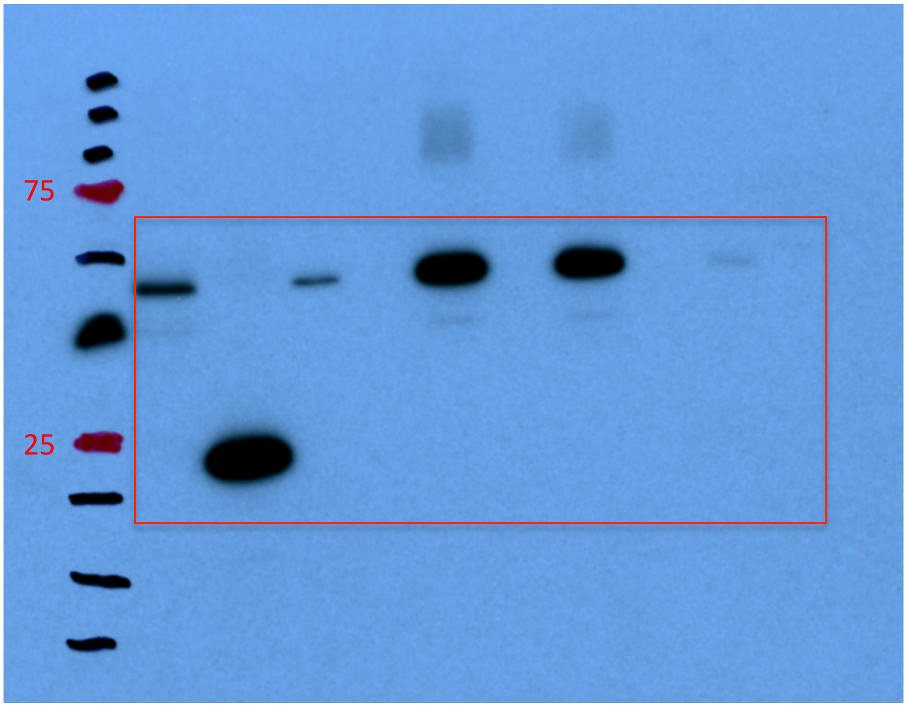

Molecular weight markers in red are 75 KD (Top) and 25 KD (Bottom)

Bottom Panel : GST-CBP incubation with E2F1 di-acetylated peptides

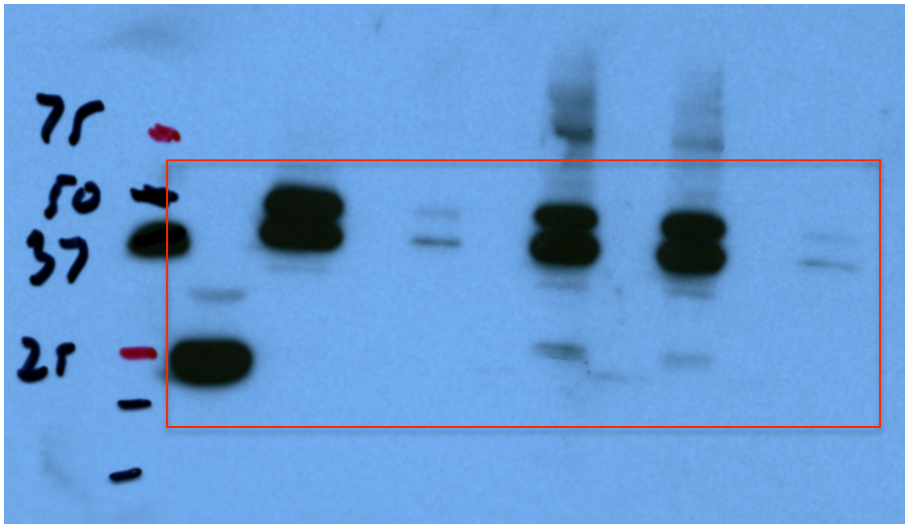

Supplementary Figure 2a

M - Molecular weight markers (KD)

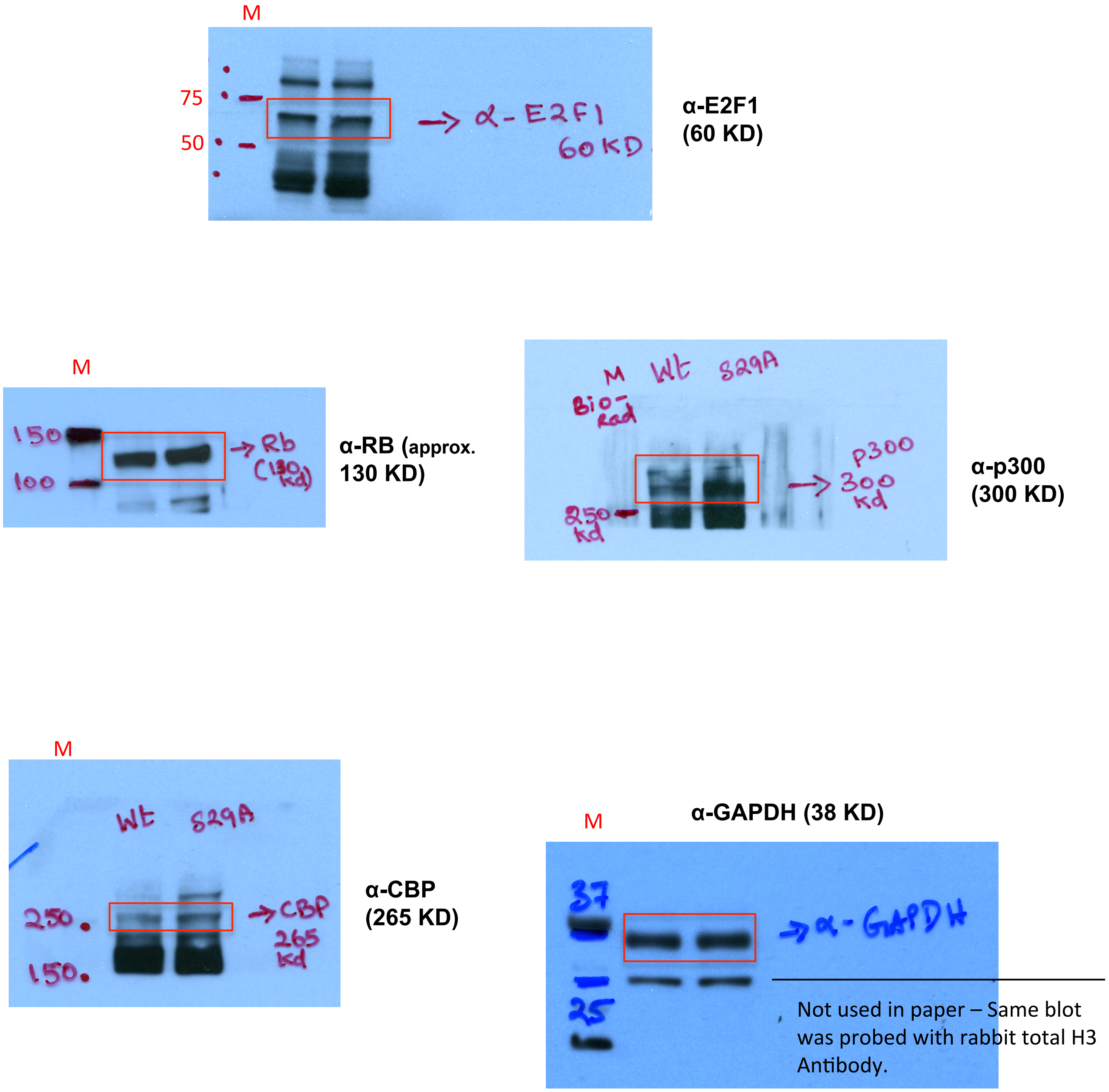

Supplementary Figure 3d

M - Molecular weight markers (KD)

M

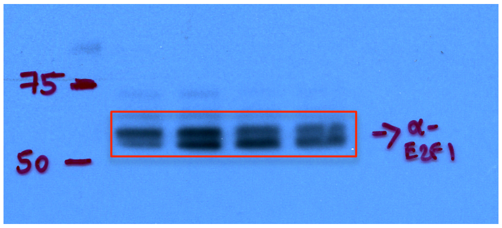

α-E2F1
(60 KD)

M

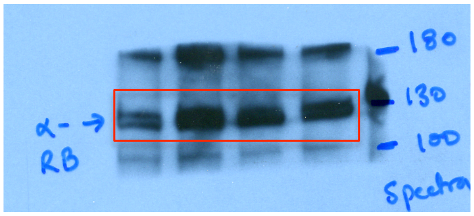

α-RB (approx.
130 KD)

M

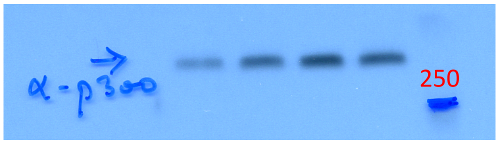

α-p300
(300 KD)

M

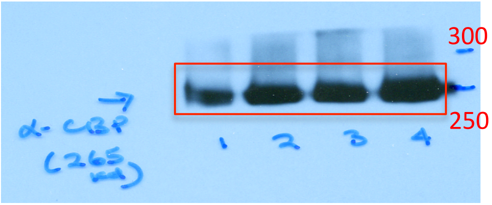

α-CBP
(265 KD)

M

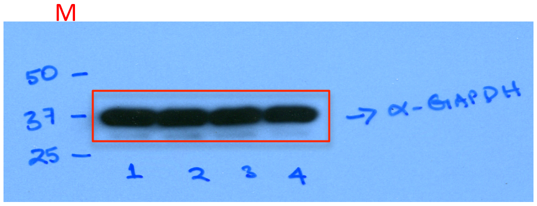

α-GAPDH
(38 KD)

Supplementary Figure 4b

Agarose Gel : Genotyping of F1 pups & F1 mouse

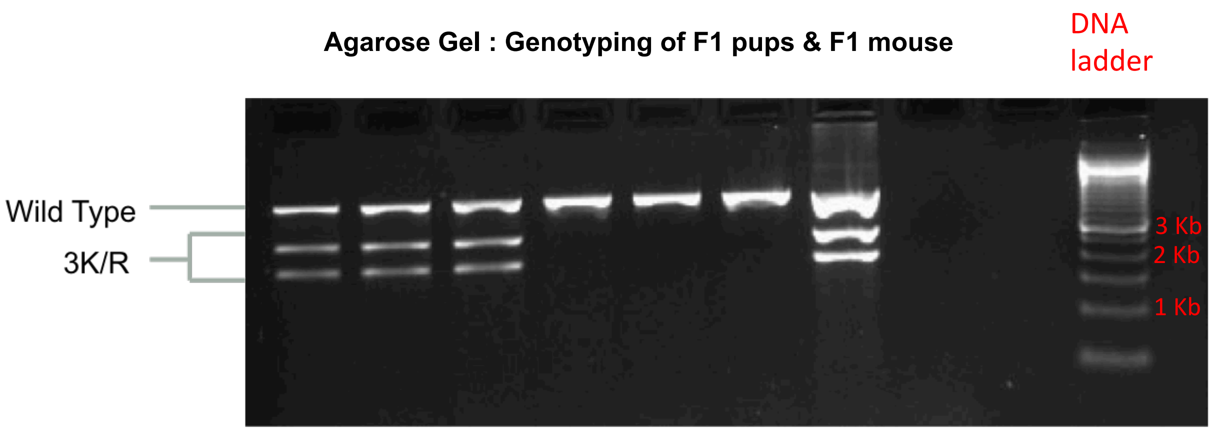

Supplementary Figure 6e

Input

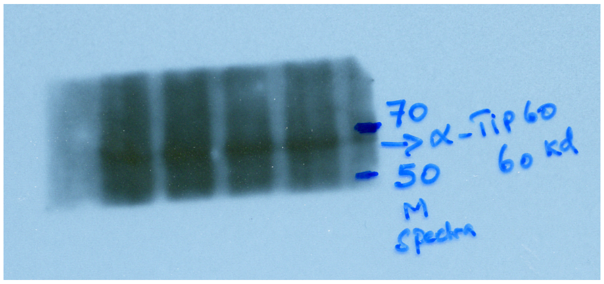

α-Tip 60 (60 KD)

α-Tip 60 (60 KD)

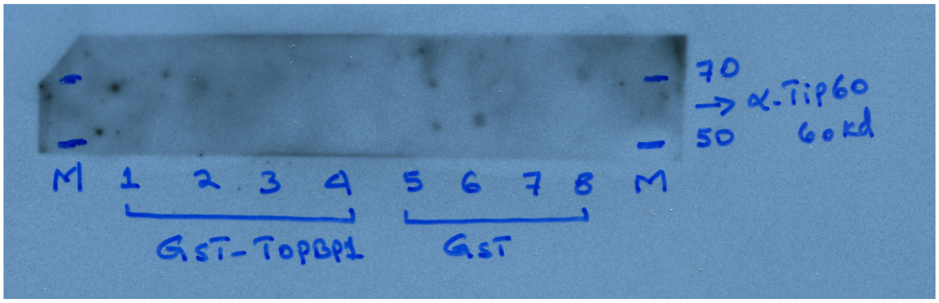

Supplementary Figure 7a

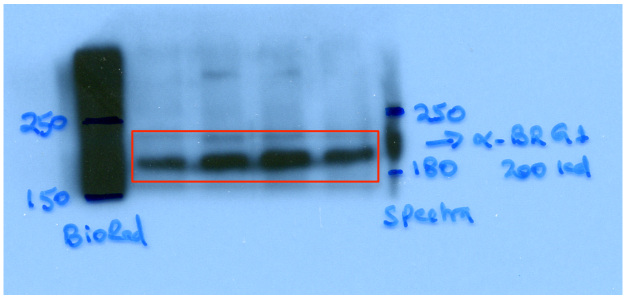

α-BRG1 (200 KD)

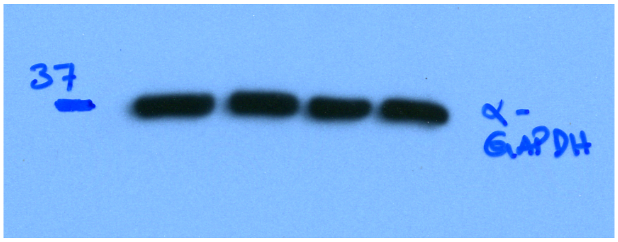

α-GAPDH
(38 KD)

Supplementary Figure 7b

α-Mre11 (81 KD)

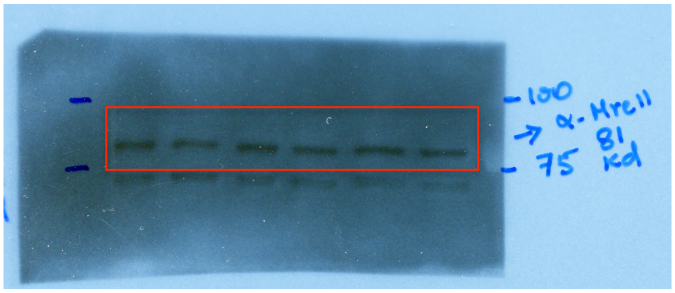

Other treatment -
Not used in paper

α-NBS1 (95 KD)

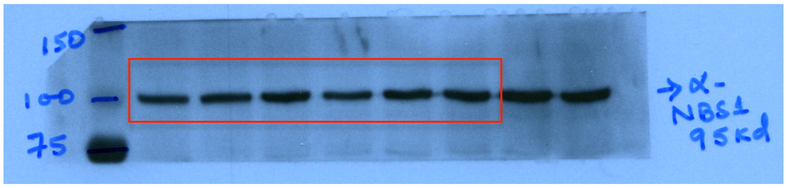

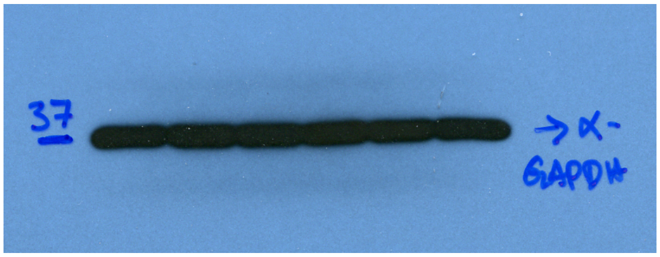

α-GAPDH
(38 KD)
